# A data-driven approach to preprocessing Illumina 450K methylation array data

**DOI:** 10.1186/1471-2164-14-293

**Published:** 2013-05-01

**Authors:** Ruth Pidsley, Chloe C Y Wong, Manuela Volta, Katie Lunnon, Jonathan Mill, Leonard C Schalkwyk

**Affiliations:** 1 Social, Genetic and Developmental Psychiatry,Institute of Psychiatry, King's College London, De Crespigny Park, London, UK; 2 University of Exeter Medical School, Exeter, UK

## Abstract

**Background:**

As the most stable and experimentally accessible epigenetic mark, DNA methylation is of great interest to the research community. The landscape of DNA methylation across tissues, through development and in disease pathogenesis is not yet well characterized. Thus there is a need for rapid and cost effective methods for assessing genome-wide levels of DNA methylation. The Illumina Infinium HumanMethylation450 (450K) BeadChip is a very useful addition to the available methods for DNA methylation analysis but its complex design, incorporating two different assay methods, requires careful consideration. Accordingly, several normalization schemes have been published. We have taken advantage of known DNA methylation patterns associated with genomic imprinting and X-chromosome inactivation (XCI), in addition to the performance of SNP genotyping assays present on the array, to derive three independent metrics which we use to test alternative schemes of correction and normalization. These metrics also have potential utility as quality scores for datasets.

**Results:**

The standard index of DNA methylation at any specific CpG site is *β* = *M*/(*M* + *U* + 100) where M and U are methylated and unmethylated signal intensities, respectively. Betas (*β*s) calculated from raw signal intensities (the default GenomeStudio behavior) perform well, but using 11 methylomic datasets we demonstrate that quantile normalization methods produce marked improvement, even in highly consistent data, by all three metrics. The commonly used procedure of normalizing betas is inferior to the separate normalization of M and U, and it is also advantageous to normalize Type I and Type II assays separately. More elaborate manipulation of quantiles proves to be counterproductive.

**Conclusions:**

Careful selection of preprocessing steps can minimize variance and thus improve statistical power, especially for the detection of the small absolute DNA methylation changes likely associated with complex disease phenotypes. For the convenience of the research community we have created a user-friendly R software package called wateRmelon, downloadable from bioConductor, compatible with the existing methylumi, minfi and IMA packages, that allows others to utilize the same normalization methods and data quality tests on 450K data.

## Background

As the most stable and experimentally accessible epigenetic mark, DNA methylation is of great interest to the epigenetics research community. The methylomic landscape across tissues, through development, and in disease pathogenesis is not yet well characterized, but a fast-growing field is exploring this methylomic variation. Illumina has recently developed the Infinium HumanMethylation microarray assay, which offers a cost-effective, high throughput method for quantitatively assessing methylation across the genome. The initial HumanMethylation27 (27K) BeadChip interrogated 27,578 CpG sites associated with 14,495 protein-coding gene promoters [1]. The more recent HumanMethylation450 (450K) BeadChip assays DNA methylation at 482,421 CpG sites, including 90% of the sites on the 27K array [2,3]. Both platforms quantify DNA methylation at single base resolution by genotyping sodium bisulfite treated DNA. The bisulfite-converted DNA is subjected to a whole-genome amplification step, followed by fragmentation and hybridization to probes on the microarray. Following hybridization, allele-specific single-base extension of the probes incorporates a fluorescent label (ddNTP) for detection. For both BeadChips the customary index of DNA methylation fraction at a specific CpG site is calculated as *β* = *M*/(*M* + *U* + *α*) where M and U are methylated and unmethylated signal intensities and *α* is an arbitrary offset (usually 100) intended to stabilize *β* values where fluorescent intensities are low. An alternative index not bounded by 0 and 1 is M = *log*_2_((*M* + *α*)/(*U* + *α*)), which is essentially equivalent to a logit transformation of *β*[4].

### Infinium humanmethylation beadchip design

In the 27K BeadChip, each CpG site is targeted by two 50 bp probes: one designed to specifically hybridize to the methylated CpG site (M); and the other to the unmethylated CpG site (U). At each CpG site single-base extension generates the same color signal for both the M and U probe. The probe design relies on the assumption that any CpG sites underlying the probe are methylated to the same extent as the target site.

The 450K BeadChip [2,3] achieves increased coverage by utilising two different probe types on each array: Infinium I (n=135,501) and Infinium II (n=350,076) probes. The Type I probes are the same design as the probes used in the 27K BeadChip, described above. The new Type II probes use just one probe per CpG locus, and employ different dye colors (green and red) to differentiate between M and U signals, respectively. The design of the Type II probes also avoids the assumption that adjacent CpG sites underlying the probe are methylated to the same extent as the target CpG site by including degenerate (R) bases at CpG sites. However the Type II probes can only include a maximum of three R bases, so Type I probes are used to assay regions of the DNA with a high density of CpG sites (for example, promoter CpG islands).

### Background

Our approach to analysis of microarray data is to try to maximize sensitivity for detection of differences between experimental groups, and accuracy of estimation of absolute methylation fraction is, if anything, secondary. In this view biases such as background are not in themselves a problem and indeed attempts to correct them, for example by subtraction of estimates derived from control probes, are undesirable because they introduce another source of variance.

**Figure 1:**
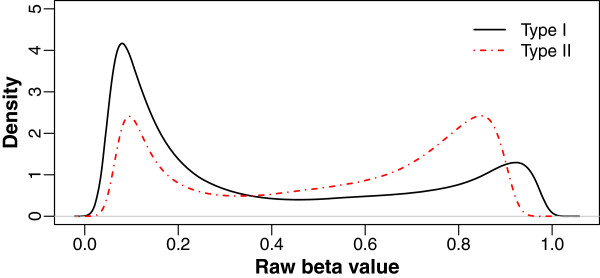
**Example of density distribution of *β * values profiled using Type I and Type II probes.**

The inclusion of two different types of chemical assay on the same array poses potential problems for data preprocessing and analysis: preprocessing methods may perform differently for the two assays; and differing distributions may make overall rankings of differentially methylated probes inaccurate. Density plots of the raw *β* values confirm that Type I and Type II probes have different distributions (Figure 1). One of our objectives is therefore to equalize this difference. Dedeurwaerder *et al*[5] found that this reflected a difference in performance between the two probe types and devised a custom transformation of the Type II *β* values to accommodate it. In the density plot of raw *β* values (Figure 1), the two peaks of the Type II probes (representing methylated and unmethylated CpG sites) are compressed toward *β*=0.5. Our insight is that a higher background in the Type II assays would explain such a difference in distribution of *β* values, because it inflates both M and U. This may be related to systematic differences in probe design such as GC content or degenerate bases, or the fact that background signal results from two colors in Type II probes and only one in Type I probes. This is also an example of how manipulations of the raw intensity can be much simpler than manipulations of *β* or M, which have complex distributions.

A number of research groups have developed preprocessing methods to accommodate the differences in signal between the two probe types [5-7]. Here, we present additional preprocessing methods to address the difference between probe types and compare our custom methods with existing methods in the literature. Our methods all operate on raw intensities and output *β* values, which can be easily transformed to M if required.

### Normalization

For the 450K Infinium HumanMethylation450 BeadChip microarray the manufacturer’s GenomeStudio software calculates *β*s from raw intensities and under default settings performs no normalization, presumably on the grounds that because the *β* readout has the total intensity in the denominator it should be insensitive to systematic differences in fluorescent intensity between samples. The *β* values are indeed fairly stable and the basic analysis method works well, particularly for the detection of large differences, for example between tissues or tumor-normal pairs [8]. However, for the investigation of subtle differences, such as those seen in common complex disorders such as schizophrenia [9,10] and diabetes [11], there is a need to ensure maximum sensitivity to detect differential DNA methylation.

A pragmatic approach to the limitations of simple ratio-based methods to calculate DNA methylation values, common in the literature [12], has been to quantile normalize *β* scores. Quantile normalization (QN) is a well established technique in gene expression analysis, where it has been shown to perform well [13]. For microarray data from multiple samples formatted as a matrix with one column per sample and one row per feature, QN is a nonlinear transformation that replaces each intensity score with the mean of the features with the same rank from each array. It is guaranteed to produce identical array-wide distributions from any data, but whether this can be achieved without losing information depends on whether the raw distributions are suitable. A potential weakness of QN is that in parts of the distribution with few values (and therefore relatively large interquantile differences), it may introduce considerable changes. The danger is that these large changes could increase the variance across samples for individual features, rather than reducing it as desired.

### Performance metrics

The suitability of QN for DNA methylation data has been assumed based on experience in the analysis of gene expression data, but there has been little systematic testing. Previous DNA methylation data analysis methods have been assessed using the DNA methylaton differences between experimental groups verified using an independent method [5]. This has the potential to be misleading as an unknown portion of the differences are artefacts. Although normalization operations manipulate the distribution of values from each sample, tests of distribution similarity between technical replicates(for example the Kolmogorov-Smirnov test used by Maksimovic *et al*[6]) are also potentially misleading because samples can be identically distributed but uncorrelated. Profile correlations are also insensitive to the potential problems of normalization because they are dominated by the majority of probes which do not show true differences, i.e. the majority of CpG sites assayed are in a fully methylated or fully unmethylated state and would change little after normalization, whilst the minority of intermediate methylation values would be susceptible to far greater changes after normalization but would be overlooked by a correlation test [14]. Because the desired result of normalization is to remove systematic errors between samples, the disappearance of batch effects is a useful indicator [12], but not sufficient as a performance indicator because it does not indicate whether true differences can still be detected. Clearly there is a need for methods that directly measure performance to predict the ability to detect true DNA methylation differences between samples. Standard, specially constructed control datasets produced by spiking samples have been influential in the gene expression field [13], but would not be as suitable for analysis of DNA methylation.

For DNA methylation we are fortunate in having natural controls: sites with a clear expectation of a defined partial methylation level. The first of these is provided by genomic imprinting. Imprinted genes are expressed monoallelically depending on parental origin, marked by allele-specific parent-of-origin dependent methylation at discrete imprinted differentially methylated regions (iDMRs). Stable iDMRs have been characterized for 25 human imprinting regions [15,16] and where array features overlap these, we would expect monoallelic (therefore 50% (*β*=0.5)) methylation.

The 450K BeadChip also features 65 control probes which assay highly-polymorphic single nucleotide polymorphisms (SNPs) rather than DNA methylation. These are included on the array to allow sample quality control to check for relatedness between individuals and enable the detection of potential sample mix-ups. The signal from these probes is expected to cluster into three distinct groups (representing the heterozygous and two homozygous groups). Although these are not DNA methylation signals, they could be used to provide an indication of the degree of technical variance between samples.

Finally, the phenomenon of X-chromosome inactivation (XCI) provides a second set of loci demonstrating predictable patterns of DNA methylation. In females, one copy of the X-chromosome is predominantly inactivated and largely methylated. Because the level of DNA methylation across active X-chromosome sites varies, we do not expect uniform X-chromosome hemi-methylation. We do, however, expect male-female differences, with females showing at least 50% methylation at CpG sites on the X-chromosome that are influenced by XCI, and males substantially less.

Armed with these potential performance metrics and some ideas about appropriate preprocessing and normalization approaches, we set out to optimize and test normalization using 11 unpublished datasets (total n=696), from our own ongoing research program (described in detail in Additional file 1).

## Results and discussion

In our ongoing work we have produced 11 450K datasets comprising samples from whole blood and four brain regions obtained from over 150 different individuals (total n=696). In exploring the data we identified and excluded a small number (less than 1%) of individual samples which were clearly technical failures based on criteria including atypical raw intensity distribution and poor correlation of *β* with other samples. Conscious of the need to minimize technical variation within datasets while retaining as much information as possible, we then explored three sets of probes which we expected to provide performance metrics that could be used to evaluate processing and normalization methods: probes in iDMRs, SNP probes, and CpG sites on the X-chromosome.

### Performance metrics

#### Imprinted differentially methylated regions

**Figure 2:**
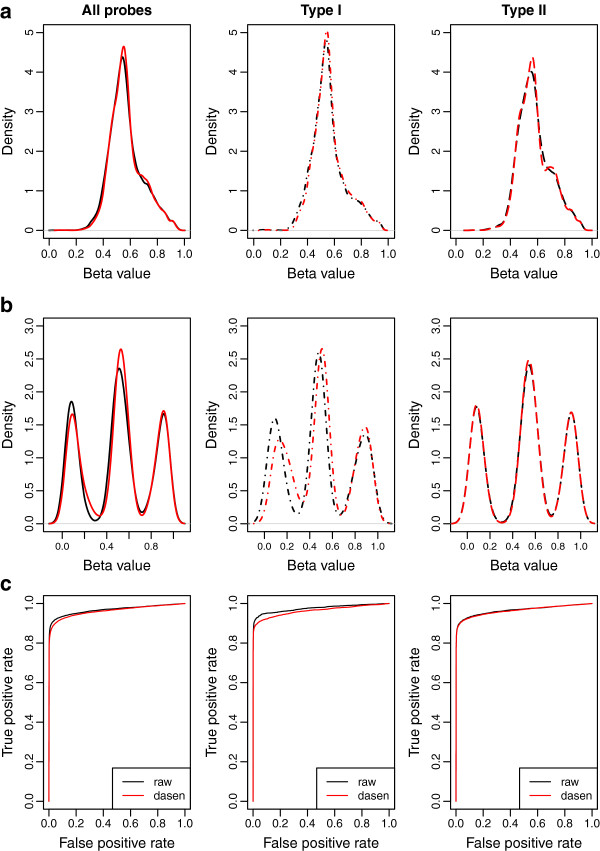
**Density plots of the *β * values generated from the raw and dasen preprocessing methods for Type I and II probes.** Plots represent the loci investigated in each of the three performance tests: **a** DMRSE, **b** GCOSE, and **c** Seabird. Black line=raw, red line=dasen.

There are 237 probes on the array that lie within a conservative set of defined iDMRs [16], and have an expected *β* value of 0.5 because they are uniparentally methylated in most tissues. From each dataset we observe a distribution of *β* for these probes with a single peak at approximately 0.5, as expected. QN produces a slightly narrower peak visible on a density plot (Figure 2a), indicating that we can detect a reduction in inter-sample variance. As a quantitative measure of this we derive a value resembling a standard error by dividing the standard deviation of the full set of DMR *β* values for the dataset by the square root of the number of samples. This measure was chosen because it is reasonably independent of the size of the dataset and it should directly predict sensitivity to detect true differences between groups. We will refer to this metric as the ‘DMRSE’ (i.e. Differentially Methylated Regions Standard Error).

#### SNP probes

There are 65 probes on the 450K BeadChip which assay highly polymorphic SNPs rather than CpG sites. As expected the *β* values for these sites cluster into three groups, depending on whether the samples are heterozygous, or homozygous at each SNP (see Figure 2b). We used k-means clustering to partition the observations into these three clusters and for each SNP returned the sum of squares and number of samples per cluster. For each SNP ideal performance (absence of technical variance) would result in zero-width peaks (i.e. sum of squares = 0). To capture this across multiple probes, we summed the 65 sum of squares and then summed the 65 number of samples per cluster (AA, AB, BB). Next we divided the summed sum of squares by the summed number of samples. In order to give a standard-error like metric we then divided these three mean-square values by the square root of the total number of samples. Finally, to simplify interpretation we combined these three measures into a single value by taking the mean, and refer to this metric as ‘GCOSE’ (Genotype Combined Standard Error).

#### X-Chromosome

The inactive X-chromosome in females is hypermethylated, and the 11,232 X-chromosome features annotated for the array show a distinctive *β* value distribution, once again centred on approximately 0.5, compared to the biphasic distribution of autosomal probes. In Cohort 1Ai for example, a t-test of each probe for sex differences gives 9,796 significant at the p=0.05 level (Bonferroni corrected for 485,577 tests), of which 8,969 correspond to X-chromosome loci. The sex difference test with this cut-off thus recovers 80% of the X-chromosome probes. 91% of the recovered differences are in X-chromosome loci. True, autosomal sex-specifically methylated loci are very small in number and potentially represent autosomal probes mapping to sequences on the sex-chromosome [17]. We can therefore conduct a Receiver Operating Characteristic (ROC) analysis, using the t-test p-value for sex difference as a predictor of X-chromosome location. The area under the curve (AUC) provides an estimate of the performance of the predictor that ranges from 0.5 for an equal chance to 1 for a perfect predictor (see Figure 2c). In order to have a metric that goes in the same direction as DMRSE and GCOSE (i.e. smaller as performance improves), we use 1−*AUC* as our metric, which we have named ‘Seabird’ (named after the auk and also the mythical bird roc).

### Preprocessing and normalization method design

Given the vast number of potential normalization and preprocessing methods, we limited our exploration to methods for which there is a rationale. The naming convention used for the different preprocessing methods is explained in full detail in Table 1. Several insights, derived from data exploration and experience, underlie our selection of methods to implement and test using the performance metrics. The first is that the primary data is methylated and unmethylated fluorescent intensities (M and U) and technical variation is likely to be more simply dealt with by adjusting these, rather than the derived *β* value, where effects may be complicated by their interaction. A further consideration here is the known potential of QN to impose large changes in those parts of a distribution with few members, which in the biphasic *β* distribution includes the potentially most interesting central part of the distribution (*β*= 0.5). Therefore in addition to testing the raw *β* values *(raw)* and QN of *β*s *(betaqn)*, we tested *β*s calculated from separately quantile normalized M and U *(naten)* (this method was introduced by Sun *et al*[12]). It could also be advantageous to separate Type I and II probes for QN (compare *nasen, dasen* with *naten, daten1*). M and U distributions differ, and so these should only be forced to the same distribution with caution, but quantile normalizing M and U against each other would have the advantage of removing dye bias variation, which is a potential problem with Type II assays, so this was performed in four methods *(nanet, nanes, danes, danet)*.

**Table 1:** Table summarising nomenclature of preprocessing methods

	**Background adjustment**	**Between-array normalization**	**Dye bias correction**
naten	n	t	n
nanet	n	n	t
nanes	n	n	s
danes	d	n	s
danet	d	n	t
danen	d	n	n
daten1	d	t	n
daten2	d	t	n
nasen	n	s	n
dasen	d	s	n

We have used a naming convention that encodes key aspects of each method. Vowels were added between the letters for ease of pronunciation. The 1st letter (column 1) indicates whether background adjustment was performed (i.e. the offset between Type I and II probe intensities added to Type I intensities), with ‘d’ indicating background adjustment and ‘n’ indicating no background adjustment. Note *daten2* is identical to *daten1* but with the addition of a linear model of Sentrix position to obtain smooth background offsets. The 3rd letter (column 2) specifies whether between-array normalization was performed (i.e. between-sample quantile normalization of M and U separately), with ‘s’ indicating between-array normalization applied to Type I and Type II probes separately, ‘t’ indicating between-array normalization applied to Type I and Type II probes together and ‘n’ indicating no between-array normalization. The 5th letter (column 3) specifies whether the dye-bias correction was performed (i.e. quantile normalization of M against U), with ‘s’ indicating dye bias correction applied to Type I and Type II probes separately, ‘t’ indicating dye bias correction applied to Type I and Type II probes together and ‘n’ indicating no dye bias correction.

Type I and Type II probes perform differently. This is not in itself a normalization issue, but it may be advantageous to minimize the differences so that the ranking of potential differentially methylated loci is more accurate [5]. This is achieved most simply by adjusting the background difference between Type I and II probes for both M and U intensities *(danes, danet, danen, daten1, daten2, dasen)*. Both methylated and unmethylated raw intensities display a characteristic peak close to zero, which differs slightly between Type I and II probes (Additional file 2). We use the position of this peak to calculate the background difference (offset) between Type I and Type II probes, and we add it to Type I intensities. Note that we do not aim to eliminate the background signal, we only seek to equalize the background signal between Type I and Type II probes. Because we observed a gradient in background in some datasets we implemented an optional linear model of Sentrix position to obtain smooth background offsets. Once again this procedure is not intended to remove the background gradient from the raw intensities, this is accomplished by subsequent QN. The objective is solely to avoid introducing additional noise while equalizing background.

### Qualitative observations on preprocessing and normalization

**Figure 3:**
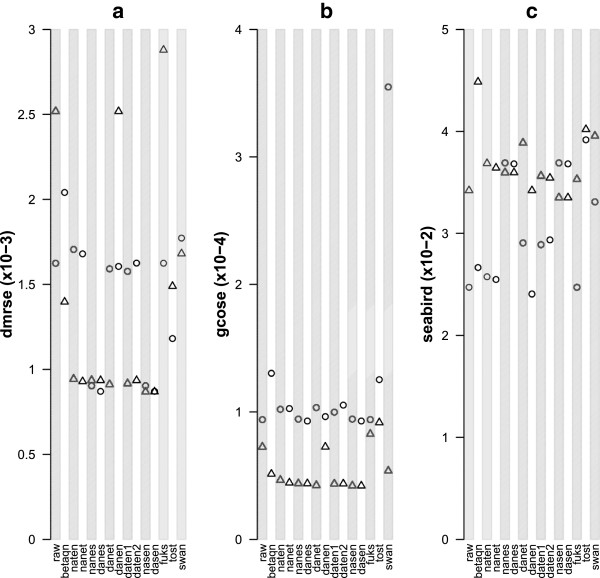
**Results of the performance tests for Cohort 1C.** All values range between 0 and 1. Lower values are indicative of a more sensitive preprocessing method. :**a**) DMRSE (x10^-3^), **b**) GCOSE (x10^-4^) and **c**) Seabird (x10^-2^). Type I probes are denoted by circles and Type II probes by triangles.

Metrics for each of the methods on one example dataset calculated separately for Type I and Type II probes are shown graphically in Figure 3. The first observation is that according to the standard error measures DMRSE and GCOSE, Type II probes perform better than Type I probes across most methods and Type I probes are more stable than Type II probes. Methods that do not use any normalization (*raw* and *Fuks*[5]) clearly perform worse for both types than those that do. Normalizing M and U is clearly better than normalizing *β* (compare *naten* with *betaqn*). Background adjustment does not introduce noticeable extra variance (compare *raw* with *danen*). Furthermore, QN of Type I and Type II intensities separately appear to reduce variance, particularly for the iDMR measures (compare *daten1* and *dasen*), but more complicated segmented QN schemes seem to be counterproductive despite embodying valuable insights about the properties of the assay (compare *Tost*[7] and *SWAN*[6] with *dasen*). Similar graphs of the three metrics for all of the datasets are presented in Additional files 3, 4 and 5.

### Systematic ranking of methods based on metrics

The test metrics were subsequently used to quantitatively determine the best performing preprocessing method (also see Additional files 3, 4, 5 and 6) using the approach detailed below and illustrated in Additional file 7. For each dataset and for Type I and Type II probes separately: 

A. Calculate the metric scores (DMRSE, GCOSE_AA, GCOSE_AB, GCOSE_BB, Seabird) for each of the 15 preprocessing methods (raw…swan)

B. For each preprocessing method take the mean of three SNP scores calculated in [A] (i.e. GCOSE_AA, GCOSE_AB, GCOSE_BB) to generate just one SNP score (GCOSE)

C. For each of the three metrics [B] rank the preprocessing methods

D. For each of the preprocessing methods calculate the mean of the three ranked metrics [C]

E. For each preprocessing method calculate the mean of mean ranks [D] across the datasets, for Type I and Type II probes separately.

F. Rank the mean of metric values across all datasets [E] to generate a final score representing the performance of each of the 15 preprocessing methods (Table 2).

**Table 2:** Overall rank scores of each preprocessing method

	**TypeI**	**TypeII**	**Average**
raw	6.5	11	8.75
betaqn	14	13	13.5
naten	12	9	10.5
nanet	11	3	7
nanes	9.5	7.5	8.5
danes	2.5	7.5	5
danet	1	6	3.5
danen	5	12	8.5
daten1	4	4	4
daten2	8	5	6.5
nasen	9.5	1.5	5.5
dasen	2.5	1.5	2
fuks	6.5	15	10.75
tost	13	14	13.5
swan	15	10	12.5

Ranks calculated from the three performance metrics applied to 11 datasets. Ranks are shown for Type I and Type II probes separately as well as averaged across both probe types. *Dasen* is shown to be the best preprocessing method across both probe types.

Using this approach, our data indicates that *dasen* is the best performing method across both probe types (Table 2). This method involves background adjustment of the methylated and unmethylated intensities. This is followed by separate QN of methylated Type I, unmethylated Type I, methylated Type II and unmethylated Type II intensities.

It is evident from the quantitative ranking method (Table 2) and figures for each individual dataset (Additional files 3, 4 and 5) that the *dasen* method performs consistently well for both the Type I and Type II probe data. Figure 2 displays the *β* values for one dataset before and after preprocessing with the *dasen* method, with the density distribution of Type I and Type II probes plotted both together and separately.

Figure 2a shows that the *dasen* method decreases the standard error across CpG sites within iDMRs, as expected (DMRSE test). Figure 2b displays the density distribution of the signal from the SNP probes (GCOSE test). While the difference in peak width is not readily visible at this scale, the effect of background adjustment on Type I probes is clearly visible. Figure 2c shows the ROC curve of true positives and false positives for predicting X-chromosome location (Seabird test). An increased area under the curve indicates improved sensitivity to detect these differences. The data is much improved for the Type II probes, which represent 72% of the probes on the array. For Cohort 1Ai for example, *dasen* gives us 296 (3%) additional sex differences at the Bonferroni-corrected p <.05 level, compared to *raw*.

### Limitations

We propose three metrics of data homogeneity that evaluate different and substantial parts of the array. For the datasets we use these give a clear picture about the optimal quantile-based normalization procedure. The possibility remains that these metrics miss some aspect of performance. We have also used a large number of experimental datasets across multiple tissues, but there may be datasets with substantially different properties that require different handling. In particular, we have only analyzed relatively homogeneous datasets derived from brain or whole blood. There may be more drastic variation in cultured cells or tumors, and we have also not extensively addressed normalization of datasets containing more than one tissue type. We expect that beyond a certain level of heterogeneity, QN procedures will be counterproductive. Our approach would be useful in evaluating this. Finally we have only tested methods based on QN. Although there is a strong consensus in favour of this method, it is possible that a different approach based, for example, on scaling, could possibly work better.

### Future directions

Rather than attempting to sample the entire universe of experiments done with this array ourselves, we are distributing what we hope is a convenient software framework to allow others to make similar tests. We do not advocate selecting or creating a custom normalization for each dataset, but it is important to find out if there are cases where the apparent best method fails.

We have implemented our functions with a simple, consistent interface that takes matrices of methylated and unmethylated intensities or *β* or M values. The source code and analysis scripts using them can be found in Additional file 8. For the convenience of users we have also created an object-oriented R software package called wateRmelon, available to download from bioConductor. In this package all of the functions mentioned above are default methods for generic functions of the same name that understand objects from the existing methylumi [18], minfi [19], and IMA [20] packages. The output from normalization methods is *β* values, but these can be conveniently transformed to M values using the *beta2m* function in the wateRmelon package.

While this work was under review, an alternative method of equalizing type I and II assay performance by applying a mixture modelling approach to *β* was published [21]. We have made the method available in the wateRmelon package. While computationally expensive, this method could potentially improve on the peak correction [5] or our background equalization method, and this could be investigated further.

## Conclusions

Currently the DNA methylation field resembles gene expression studies of a decade ago: it is technology-limited and characterized by small sample sizes, diverse techniques and variable statistical standards [22]. Fortunately the field can benefit from the development work invested over the last ten years in gene expression and genotyping technology, and we can quickly develop and test sophisticated analysis methods. We have used 11 datasets to investigate 15 different methods of correction and normalization. We then evaluated each method using features on the array that assay known sites of differential methylation or genotype. The results of our tests reveal that a combination of background adjustment and between-array quantile normalization is optimal for data processing, to allow the detection of differential methylation between samples.

## Methods

### Samples

11 datasets were used to evaluate our preprocessing pipeline (Additional file 1). Seven of the datasets were obtained from one cohort of post-mortem brain samples from the MRC London Brain Bank for Neurodegenerative Diseases (http://www.kcl.ac.uk/iop/ depts/cn/research/mrclondonbrainbank.aspx): cerebellum (1Ai) (n=91), rescan of cerebellum data (1Aii) (n=36), frontal cortex (1B) (n=89), entorhinal cortex (1C) (n=93), superior temporal gyrus (1D) (n=94), whole blood (1E) (n=95), frontal cortex and entorhinal cortex (1BC) (n=46), cerebellum and superior temporal gyrus (1AD) (n=47). Two of the datasets were obtained from a separate cohort from the London Brain Bank for Neurodegenerative Diseases: cerebellum (2A) (n=42), frontal cortex (2B) (n=43). The final dataset was obtained from DNA from the Autism Tissue Program (http://www.autismtissueprogram.org/): cerebellum (3A) (n=18).

### 450K methylation beadchip analysis

500 ng of genomic DNA from each sample was treated with sodium bisulfite in duplicate, using the EZ96 DNA methylation kit (Zymo Research, CA, USA) following the manufacturer’s standard protocol. Genome-wide DNA methylation was assessed using the Illumina Infinium HumanMethylation450 BeadChip (Illumina Inc, CA, USA) according to manufacturer’s instructions. Illumina GenomeStudio software was used to extract the raw signal intensities of each probe (without background correction or normalization).

### Statistical analysis

All computations and statistical analyses were performed using R 2.15.0 [23] and Bioconductor 2.12 [24]. Signal intensities were imported into R using the methylumi package [18] as a methylumi object. Initial quality control checks were performed using functions in the methylumi package to assess concordance between reported and genotyped gender. Non-CpG SNP probes on the array were also used to confirm that multiple tissues were sourced from the same individual where expected. Comparative analysis was performed using R scripts provided in Additional file 8. For the convenience of users we have also packaged the functions into an R package: wateRmelon, which is available from the bioConductor repository from version 2.12 (for R version 3.0 and higher). The functions in the package are all generic and methods are provided for the objects produced by the packages methylumi [18], minfi [19], and IMA [20], as well as wrappers for the methods of Dedeurwaerder *et al*[5] A number of packages from CRAN and bioConductor were used: quantile normalization using limma [25]; data handling using methylumi [18] and minfi [19]; and performance assessment using ROCR [26]. All of the data used in this publication has been deposited in GEO (series accession GSE43414).

### Ethical approval

Epigenomic profiling on post-mortem brain tissue was approved by the UK National Health Service (NHS) National Research Ethics Service (NRES) (reference number: 10/H0808/114). Tissue obtained from the Medical Research Council (MRC) Brainbank for Neurodegerative Diseases was consented fully prior to death and approved by the NHS RES (reference number: 08/MRE09/38). The Human Tissue Authority (HTA) license number for the brain bank is 12293.

## Abbreviations

iDMR: imprinted differentially methylated region; SNP: single nucleotide polymorphism; XCI: X-chromosome inactivation; ROC: Receiver Operating Characteristic; AUC: area under the curve; QN: quantile normalization; M: methylated signal.

## Competing interests

The authors declare that they have no competing interests.

## Authors’ contributions

JM and LCS conceived the experiments, and RP, CCYW, KL, and MV executed them. LCS, RP and CCYW designed the analysis methods, LCS implemented them and LCS, RP and CCYW ran the analyses. LCS, RP and CCYW wrote the paper with contributions from all authors. All authors read and approved the final manuscript.

## Supplementary Material

Additional file 1 Summary of cohorts used in this study. Click here for file

Additional file 2 **Density plots of the methylated (M) and unmethylated (U) raw signal intensities.** Type I and II probes plotted separately, with maximum signal peak heights represented by dotted lines. Horizontal red line represents offset between the maximum peak height of probe Types I and II. The offset is added to Type I assay intensities to equalize background in the methods whose names begin with ‘d’. Click here for file

Additional file 3 **Results of the DMRSE performance tests for all remaining datasets (x10-3 scale is used on the y-axis).** Lower values are indicative of a more sensitive preprocessing method. Type I probes are denoted by circles and Type II probes by triangles. Click here for file

Additional file 4 **Results of the GCOSE performance tests for all remaining datasets (x10-4 scale is used on the y-axis).** Lower values are indicative of a more sensitive preprocessing method. Type I probes are denoted by circles and Type II probes by triangles. Click here for file

Additional file 5 **Results of the Seabird performance tests for all remaining datasets (x10-2 scale is used on the y-axis).** Cohort 3A is absent because all samples were male, making the Seabird test redundant. Lower values are indicative of a more sensitive preprocessing method. Type I probes are denoted by circles and Type II probes by triangles. Click here for file

Additional file 6 **Results of the GCOSE performance tests split by genotype group for all datasets (x10-4 scale is used on the y-axis).** Lower values are indicative of a more sensitive preprocessing method. Type I probes are denoted by circles and Type II probes by triangles. This shows that the relative performance of our custom methods perform consistently across the range of betas, and that the tost method performs worst in the mid-range while swan does worst at the extremes. Click here for file

Additional file 7 Illustration of method ranking procedure. Click here for file

Additional file 8 Source code and analysis scripts used in this paper and the wateRmelon R package. Click here for file

## References

[B1] Bibikova M Le J Barnes B Genome-wide DNA methylation profiling using Infinium assay Epigenomics 2009 1 177 200 [http://www.ingentaconnect.com/content/fm/epi/2009/00000001/00000001/art00019] 10.2217/epi.09.14 22122642

[B2] Bibikova M Barnes B Tsan C Ho V Klotzle B Le JM Delano D Zhang L Schroth GP Gunderson KL Fan JB Shen R High density DNA methylation array with single CpG site resolution Genomics 2011 98 4 288 295 [http://www.ncbi.nlm.nih.gov/pubmed/21839163] 10.1016/j.ygeno.2011.07.007 21839163

[B3] Sandoval J Heyn Ha Moran S Serra-Musach J Pujana MA Bibikova M Esteller M Validation of a DNA methylation microarray for 450,000 CpG sites in the human genome Epigenetics 2011 6 6 692 702 [http://www.landesbioscience.com/journals/epigenetics/article/16196/] 10.4161/epi.6.6.16196 21593595

[B4] Du P Zhang X Huang CC Jafari N Kibbe WA Hou L Lin SM Comparison of Beta-value and M-value methods for quantifying methylation levels by microarray analysis BMC Bioinformatics 2010 11 587 10.1186/1471-2105-11-587 21118553 PMC3012676

[B5] Dedeurwaerder S Defrance M Calonne E Sotiriou C Fuks F Evaluation of the Infinium Methylation 450K technology Epigenetics 2011 3 6 771 784 10.2217/epi.11.105 22126295

[B6] Maksimovic J Gordon L Oshlack A SWAN: Subset quantile within-array normalization for Illumina Infinium HumanMethylation450 BeadChips Genome Biol 2012 13 6 R44 [http://www.ncbi.nlm.nih.gov/pubmed/22703947] 10.1186/gb-2012-13-6-r44 22703947 PMC3446316

[B7] Touleimat N Tost J Complete pipeline for Infinium ^®^, Human Methylation 450K BeadChip data processing using subset quantile normalization for accurate DNA methylation estimation Epigenomics 2012 4 325 341 [http://www.futuremedicine.com/doi/abs/10.2217/epi.12.21] 10.2217/epi.12.21 22690668

[B8] Carén H Djos A Nethander M Sjöberg RM Kogner P Enström C Nilsson S Martinsson T Identification of epigenetically regulated genes that predict patient outcome in neuroblastoma BMC Cancer 2011 11 66 [http://www.pubmedcentral.nih.gov/articlerender.fcgi?artid=3045360∖&tool=pmcentrez∖&rendertype=abstract] 10.1186/1471-2407-11-66 21314941 PMC3045360

[B9] Dempster EL Pidsley R Schalkwyk LC Owens S Georgiades A Kane F Kalidindi S Picchioni M Kravariti E Toulopoulou T Murray RM Mill J Disease-associated epigenetic changes in monozygotic twins discordant for schizophrenia and bipolar disorder Human Mol Genet 2011 20 24 4786 4796 10.1093/hmg/ddr416 21908516 PMC3221539

[B10] Kinoshita M Numata S Tajima A Shimodera S Ono S Imamura A Iga JI Watanabe S Kikuchi K Kubo H Nakataki M Sumitani S Imoto I Okazaki Y Ohmori T DNA methylation signatures of peripheral leukocytes in Schizophrenia Neuromolecular Med 2012 [http://www.ncbi.nlm.nih.gov/pubmed/22961555] 10.1007/s12017-012-8198-6 22961555

[B11] Rakyan VK Beyan H Down Ta Hawa MI Maslau S Aden D Daunay A Busato F Mein Ca Manfras B Dias KRM Bell CG Tost J Boehm BO Beck S Leslie RD Identification of type 1 diabetes-associated DNA methylation variable positions that precede disease diagnosis PLoS Genet 2011 7 9 e1002300 [http://www.pubmedcentral.nih.gov/articlerender.fcgi?artid=3183089∖&tool=pmcentrez∖&rendertype=abstract] 10.1371/journal.pgen.1002300 21980303 PMC3183089

[B12] Sun Z Chai HS Wu Y White WM Donkena KV Klein CJ Garovic VD Therneau TM Kocher JPa Batch effect correction for genome-wide methylation data with Illumina Infinium platform BMC Med Genomics 2011 4 84 [http://www.pubmedcentral.nih.gov/articlerender.fcgi?artid=3265417∖&tool=pmcentrez∖&rendertype=abstract] 10.1186/1755-8794-4-84 22171553 PMC3265417

[B13] Irizarry Ra Hobbs B Collin F Beazer-Barclay YD Antonellis KJ Scherf U Speed TP Exploration, normalization, and summaries of high density oligonucleotide array probe level data Biostatistics (Oxford, England) 2003 4 2 249 264 [http://www.ncbi.nlm.nih.gov/pubmed/12925520] 10.1093/biostatistics/4.2.249 12925520

[B14] Roessler J Ammerpohl O Gutwein J Hasemeier B Anwar SL Kreipe H Lehmann U Quantitative cross-validation and content analysis of the 450k DNA methylation array from Illumina, Inc BMC Res Notes 2012 5 210 [http://www.pubmedcentral.nih.gov/articlerender.fcgi?artid=3420245∖&tool=pmcentrez∖&rendertype=abstract] 10.1186/1756-0500-5-210 22546179 PMC3420245

[B15] Schulz R Woodfine K Menheniott TR Bourc’his D Bestor T Oakey RJ WAMIDEX: a web atlas of murine genomic imprinting and differential expression Epigenetics 2008 3 2 89 96 [http://www.landesbioscience.com/journals/epi/article/5900/] 10.4161/epi.3.2.5900 18398312 PMC2492384

[B16] Schulz R Wamidex, accessed 26 Jan2012 [https://atlas.genetics.kcl.ac.uk/]

[B17] Chen Ya Choufani S Ferreira JC Grafodatskaya D Butcher DT Weksberg R Sequence overlap between autosomal and sex-linked probes on the Illumina HumanMethylation27 microarray Genomics 2011 97 4 214 222 [http://www.ncbi.nlm.nih.gov/pubmed/21211562] 10.1016/j.ygeno.2010.12.004 21211562

[B18] Davis S Du P Bilke S Triche JrT Bootwalla M Methylumi: Handle Illumina Methylation Data 2012 R package version 2.2.0

[B19] Hansen KD Aryee M Minfi: Analyze Illumina’s 450k Methylation Arrays R package version 1.2.0

[B20] Wang D Yan L Hu Q Sucheston LE Higgins MJ Ambrosone CB Johnson CS Smiraglia DJ Liu S IMA: an R package for high-throughput analysis of Illumina’s 450K Infinium methylation data Bioinformatics (Oxford, England) 2012 28 5 729 730 [http://www.pubmedcentral.nih.gov/articlerender.fcgi?artid=3289916∖&tool=pmcentrez∖&rendertype=abstract] 10.1093/bioinformatics/bts013 PMC3289916 22253290

[B21] Teschendorff AE Marabita F Lechner M Bartlett T Tegner J Gomez-Cabrero D Beck S A Beta-Mixture quantile normalisation method for correcting probe design bias in Illumina Infinium 450k DNA methylation data Bioinformatics 2012 29 2 189 96 [http://www.ncbi.nlm.nih.gov/pmc/articles/PMC3546795/] 23175756 10.1093/bioinformatics/bts680 PMC3546795

[B22] Heijmans BT Mill J Commentary: The seven plagues of epigenetic epidemiology Int J Epidemiol 2012 41 74 78 [http://www.ncbi.nlm.nih.gov/pubmed/22269254] 10.1093/ije/dyr225 22269254 PMC3304528

[B23] R Development Core Team Vienna: R Foundation for Statistical Computing; 2012.: ISBN 3-900051-07-0 [http://www.R-project.org/]

[B24] Gentleman RC Carey VJ Bates DM Bioconductor: open software development for computational biology and bioinformatics Genome Biol 2004 5 R80 [http://genomebiology.com/2004/5/10/R80] 10.1186/gb-2004-5-10-r80 PMC545600 15461798

[B25] Smyth GK Gentleman R CareyVDudoit S Limma: linear models for microarray data Bioinformatics and Computational Biology Solutions using R and Bioconductor 2005 New York: Springer 397 420

[B26] Sing T Sander O Beerenwinkel N Lengauer T 2009 R package version 1.0-4 [http://CRAN.R-project.org/package=ROCR.] 10.1093/bioinformatics/bti623 16096348

