# Supplementary material for: A data-driven approach to preprocessing Illumina 450K methylation array data
Source: BMC Genomics. 2013 May 1;14:293. doi: 10.1186/1471-2164-14-293 (PMC3769145; doi:10.1186/1471-2164-14-293)
Supplement: Additional file 1 — Summary of cohorts used in this study. [file 1471-2164-14-293-S1.pdf]

Cohort	Sample Type	Total N	Total number of arrays	Gender		
				Female	Male	NA
Cohort 1Ai	Cerebellum	91	8	55	36	
Cohort 1Aii	Cerebellum	36	3	21	13	2
Cohort 1B	Frontal cortex	89	8	56	33	
Cohort 1C	Entorhinal cortex	93	8	59	34	
Cohort 1D	Superior temporal gyrus	94	8	57	37	
Cohort 1E	Whole blood	95	8	53	28	14
Cohort 1AD	Cerebellum, Superior temporal gyrus	47	4	11, 13	12, 11	
Cohort 1BC	Frontal cortex, Entorhinal cortex	46	4	11,7	16,12	
Cohort 2A	Cerebellum	42	4	14	28	
Cohort 2B	Frontal cortex	43	4	15	28	
Cohort 3A	Cerebellum	18	2	0	18	
