# Supplementary figures and images for: A data-driven approach to preprocessing Illumina 450K methylation array data
Source: BMC Genomics. 2013 May 1;14:293. doi: 10.1186/1471-2164-14-293 (PMC3769145; doi:10.1186/1471-2164-14-293)

## Methylated Signal Intensities

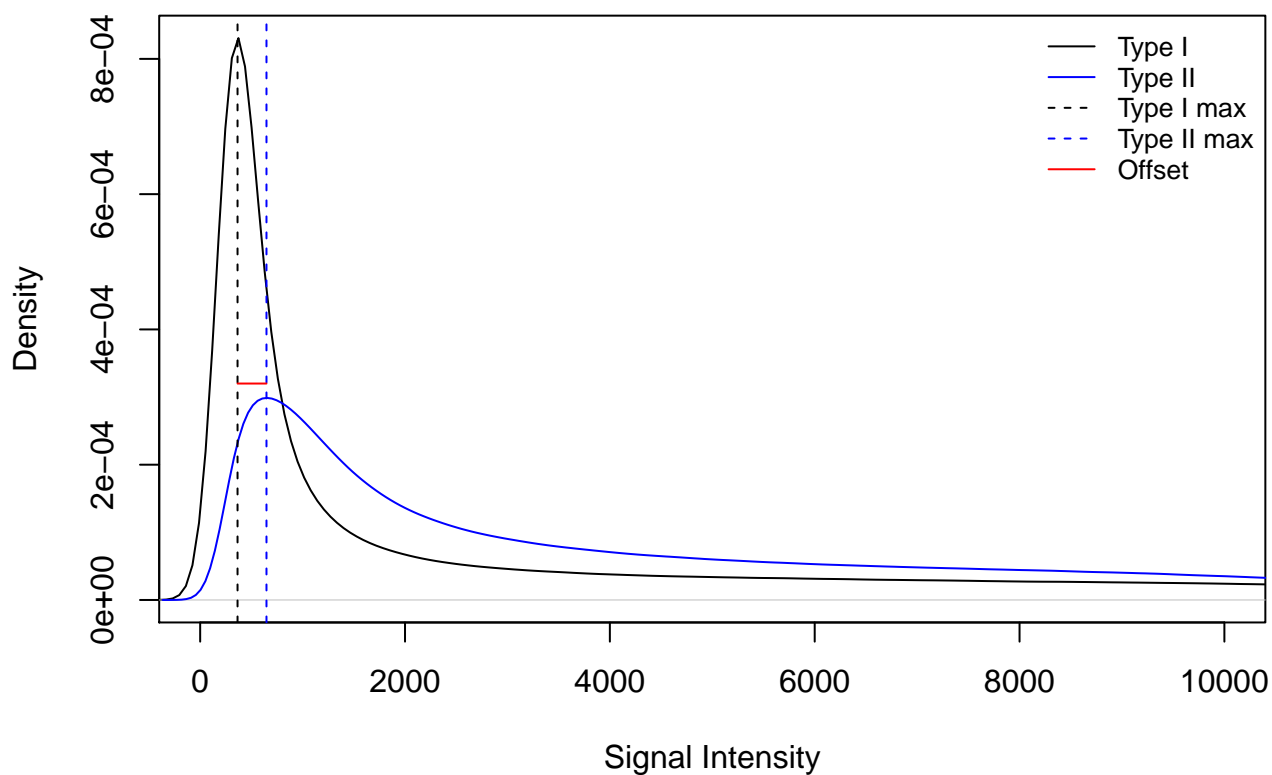

## Unmethylated Signal Intensities

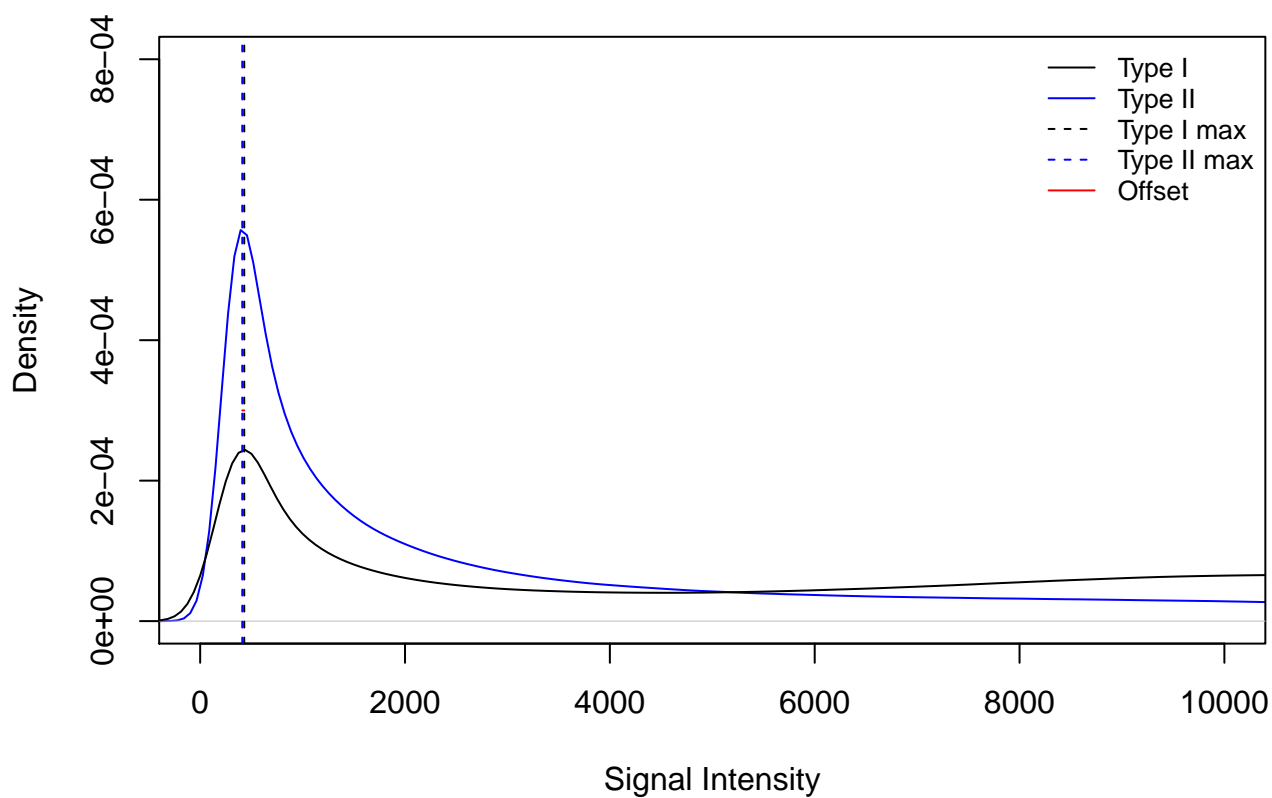

Supplement: Additional file 2 — Density plots of the methylated (M) and unmethylated (U) raw signal intensities. Type I and II probes plotted separately, with maximum signal peak heights represented by dotted lines. Horizontal red line represents offset between the maximum peak height of probe Types I and II. The offset is added to Type I assay intensities to equalize background in the methods whose names begin with ‘d’. [file 1471-2164-14-293-S2.pdf]

## Cohort\_1B

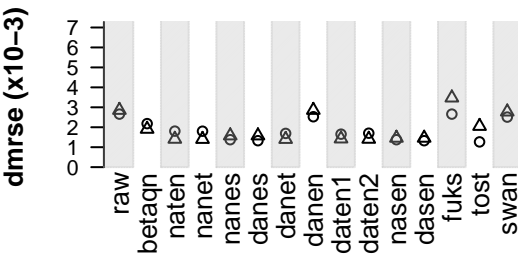

## Cohort\_1D

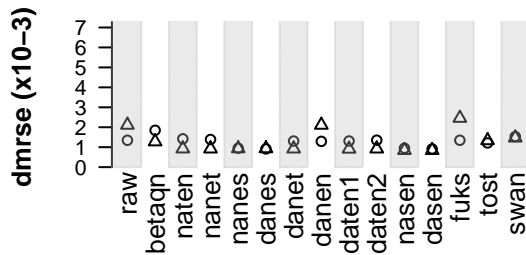

## Cohort\_1Ai

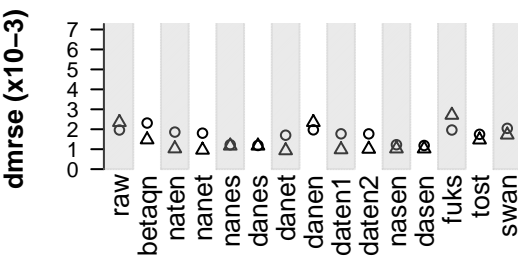

### Cohort\_1BC

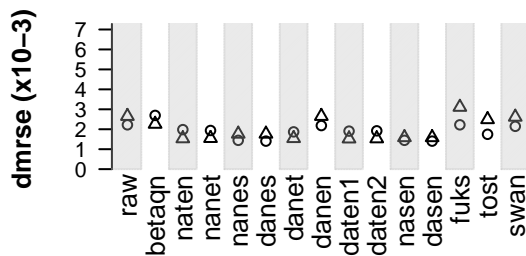

## Cohort\_1AD

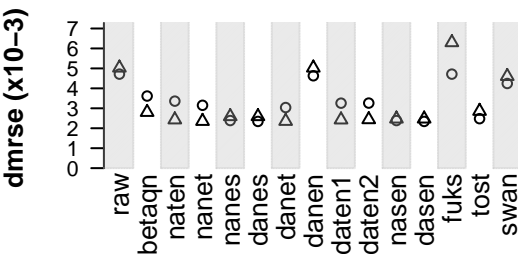

**Cohort\_1Aii**

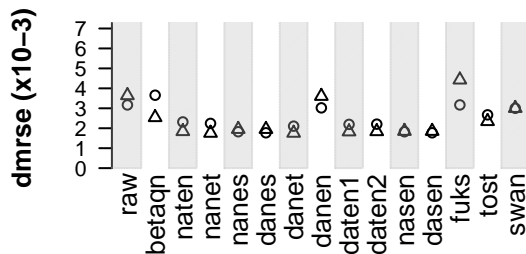

dmrse (x10-3)

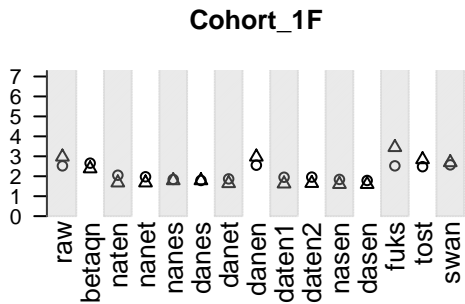

dmrse (x10-3)

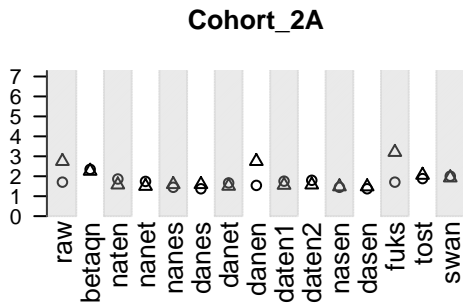

dmrse (x10-3)

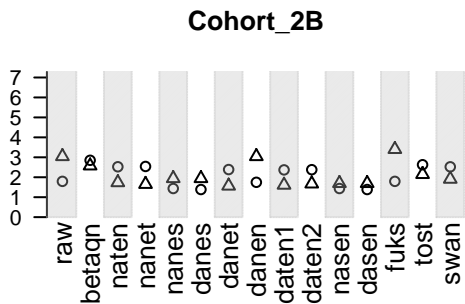

dmrse (x10-3)

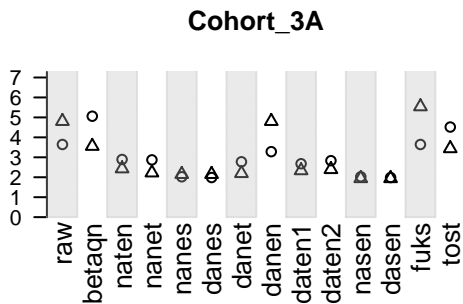

Supplement: Additional file 3 — Results of the DMRSE performance tests for all remaining datasets (x10-3 scale is used on the y-axis). Lower values are indicative of a more sensitive preprocessing method. Type I probes are denoted by circles and Type II probes by triangles. [file 1471-2164-14-293-S3.pdf]

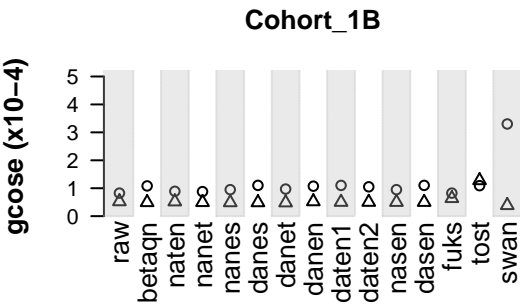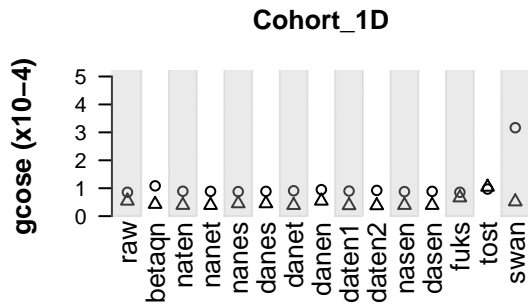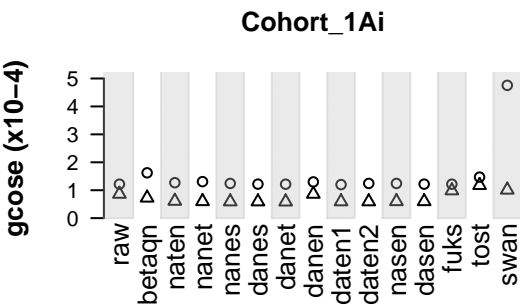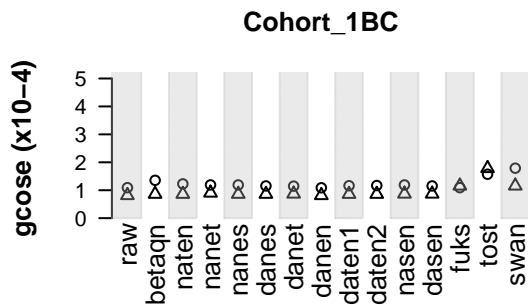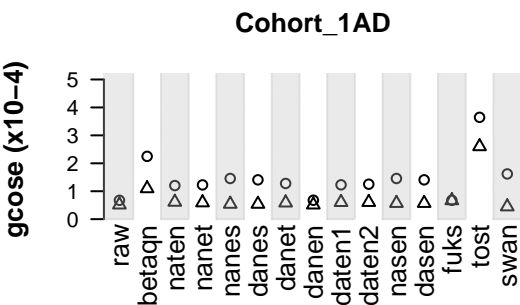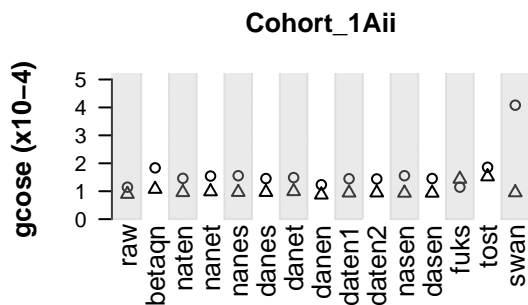

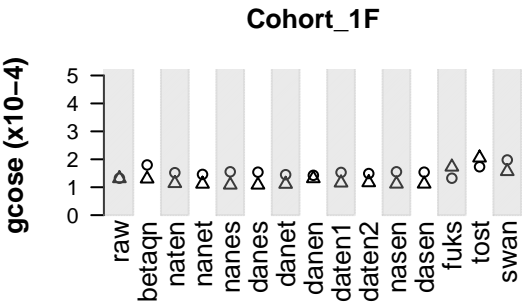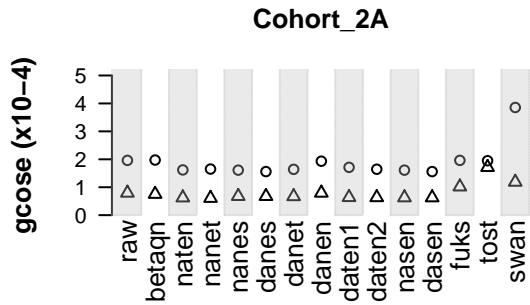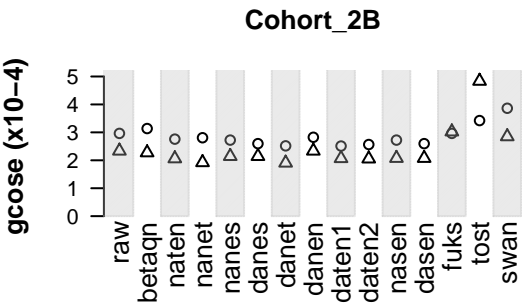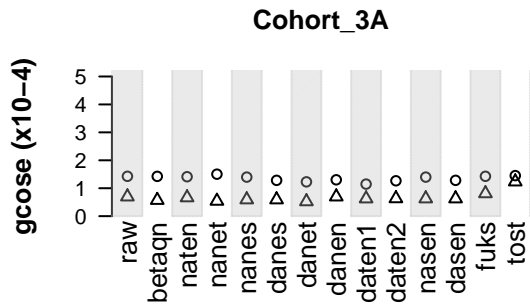

Supplement: Additional file 4 — Results of the GCOSE performance tests for all remaining datasets (x10-4 scale is used on the y-axis). Lower values are indicative of a more sensitive preprocessing method. Type I probes are denoted by circles and Type II probes by triangles. [file 1471-2164-14-293-S4.pdf]

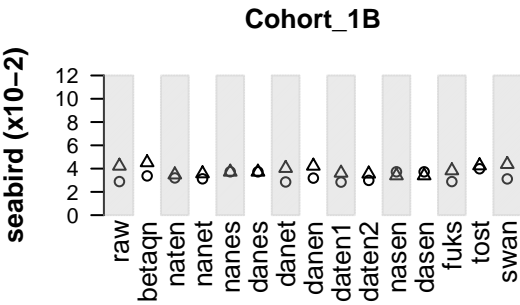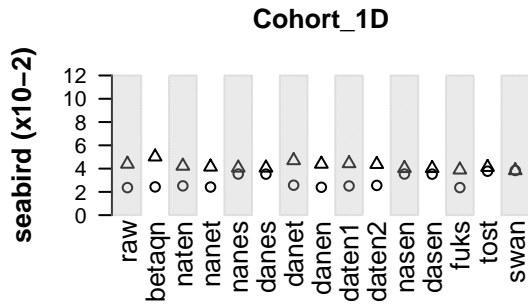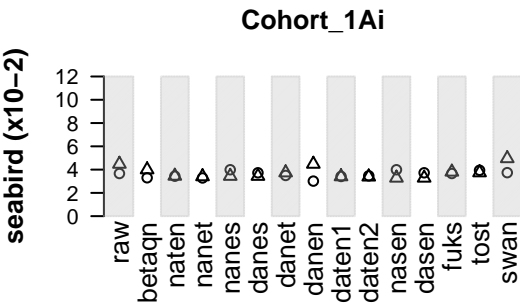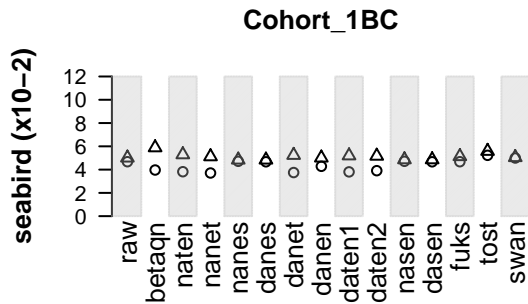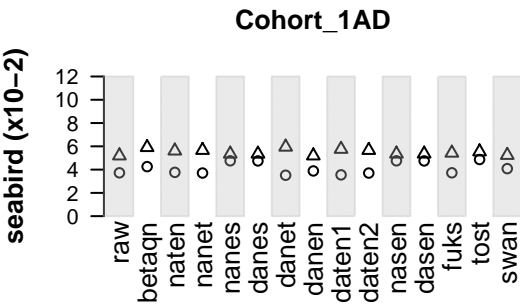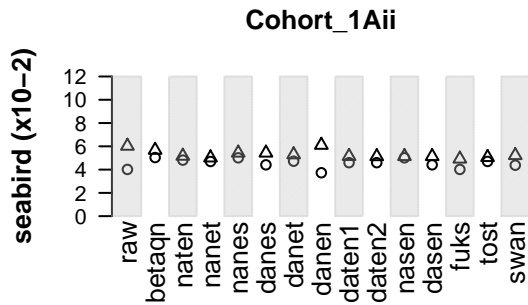

seabird (x10-2)

Cohort\_1F

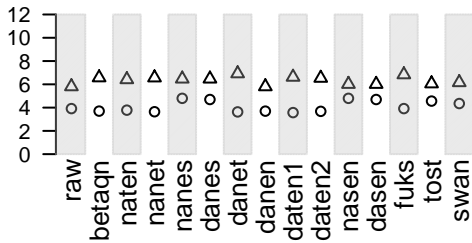

seabird (x10-2)

Cohort\_2A

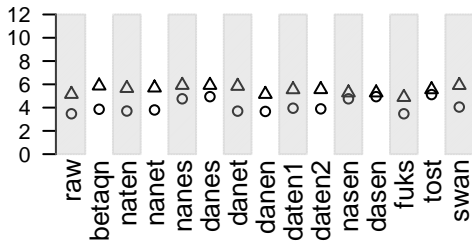

seabird (x10-2)

Cohort\_2B

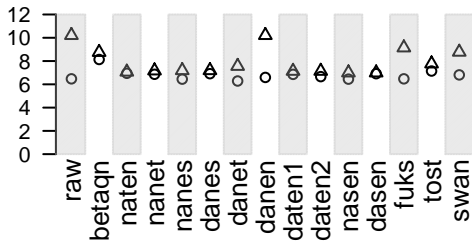

Supplement: Additional file 5 — Results of the Seabird performance tests for all remaining datasets (x10-2 scale is used on the y-axis). Cohort 3A is absent because all samples were male, making the Seabird test redundant. Lower values are indicative of a more sensitive preprocessing method. Type I probes are denoted by circles and Type II probes by triangles. [file 1471-2164-14-293-S5.pdf]

## Cohort\_1B

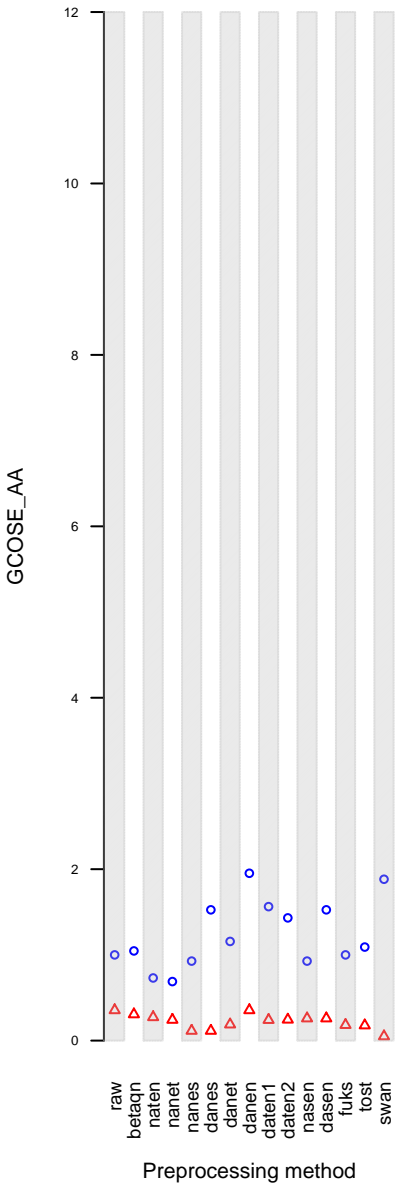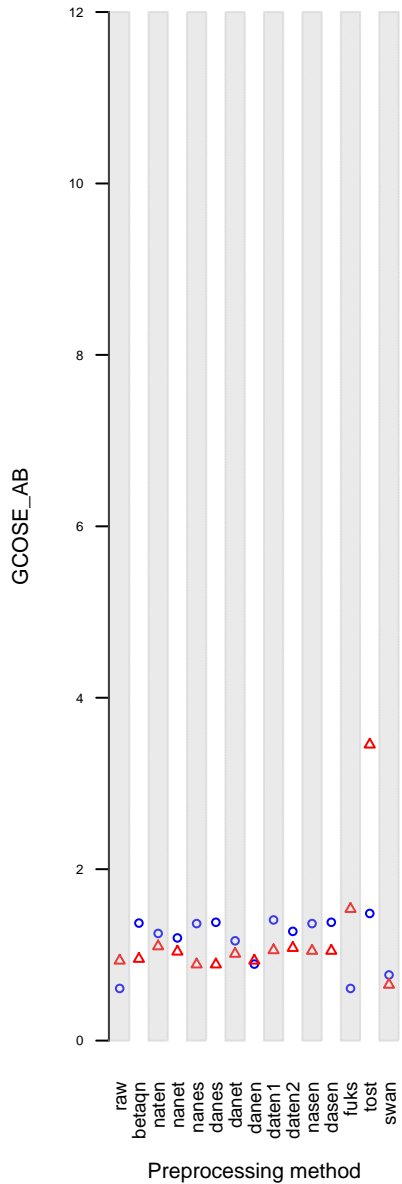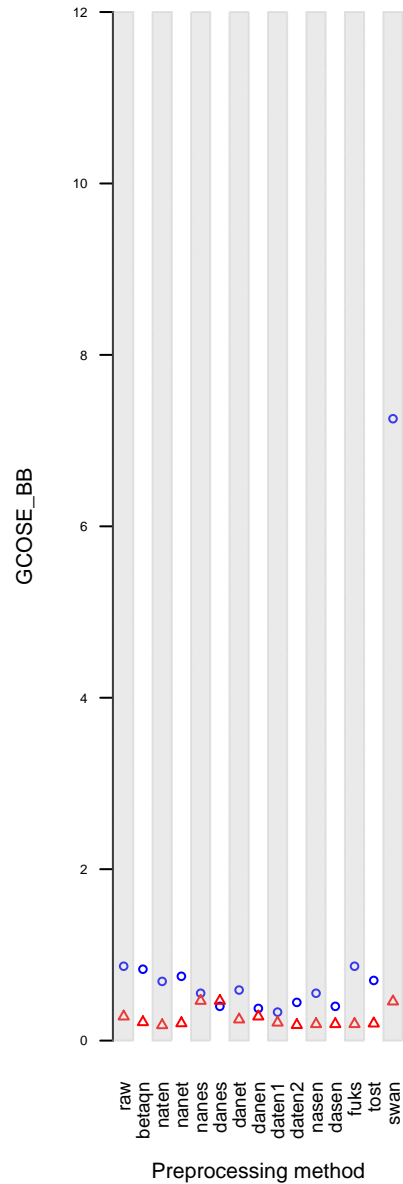

# Cohort\_1C

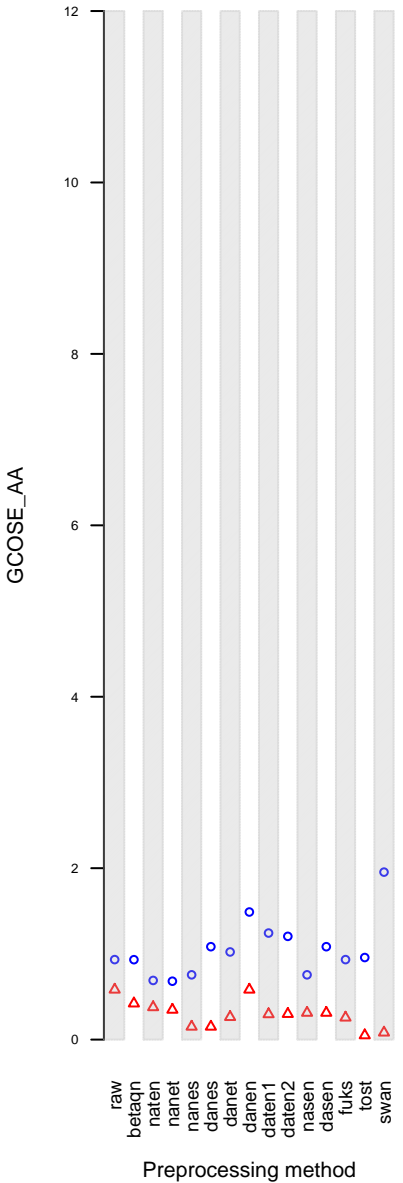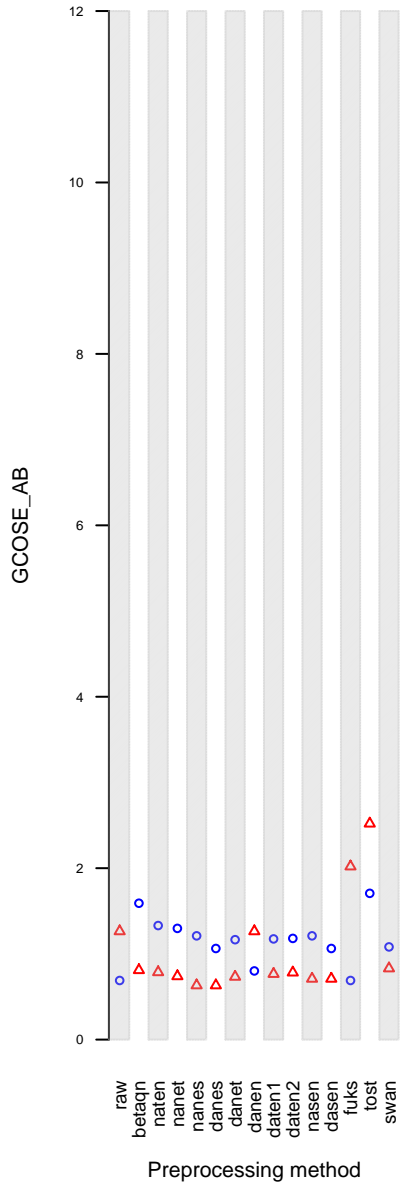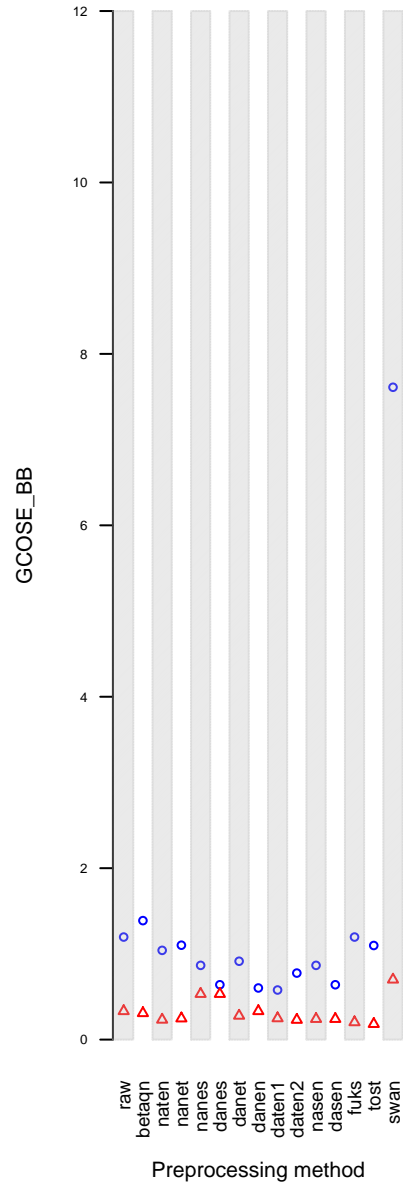

## Cohort\_1D

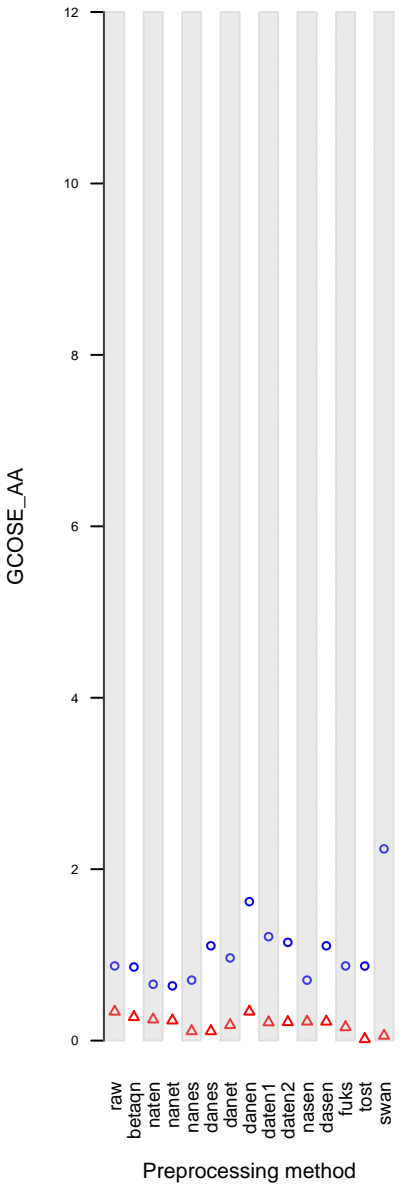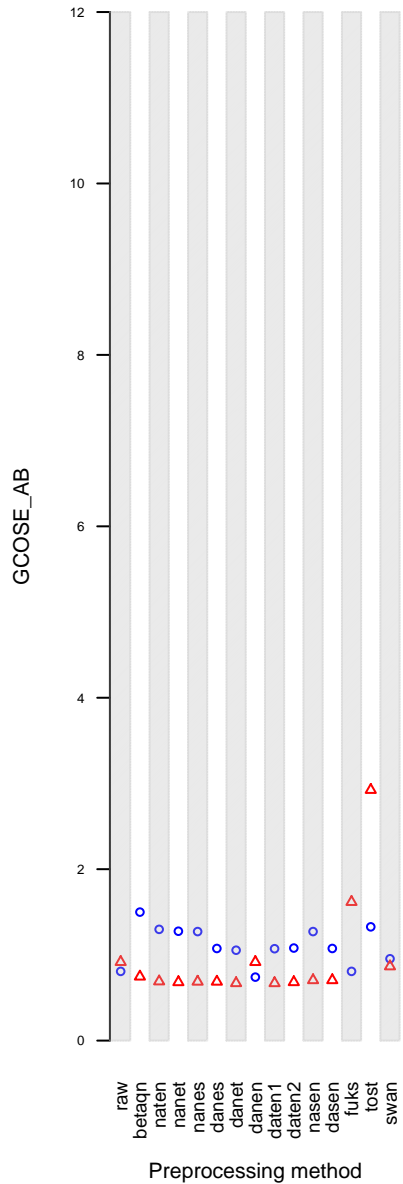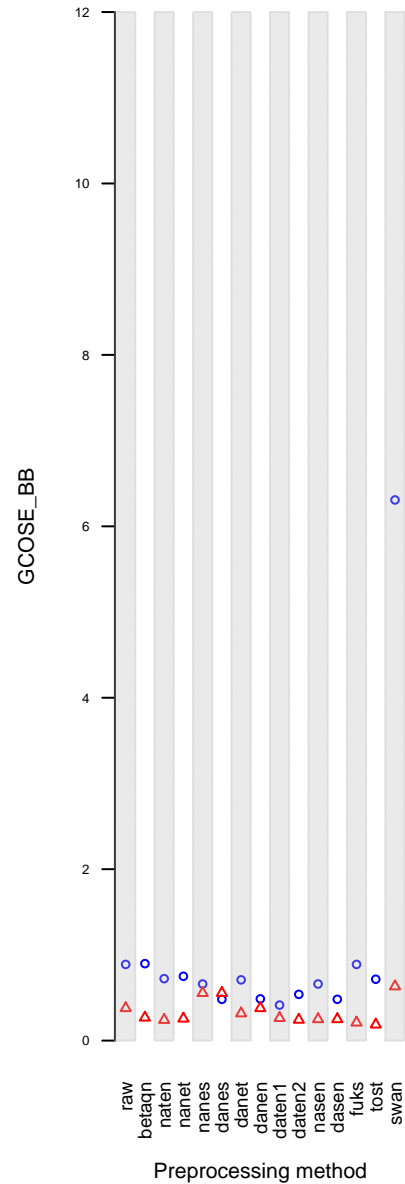

# Cohort\_1Ai

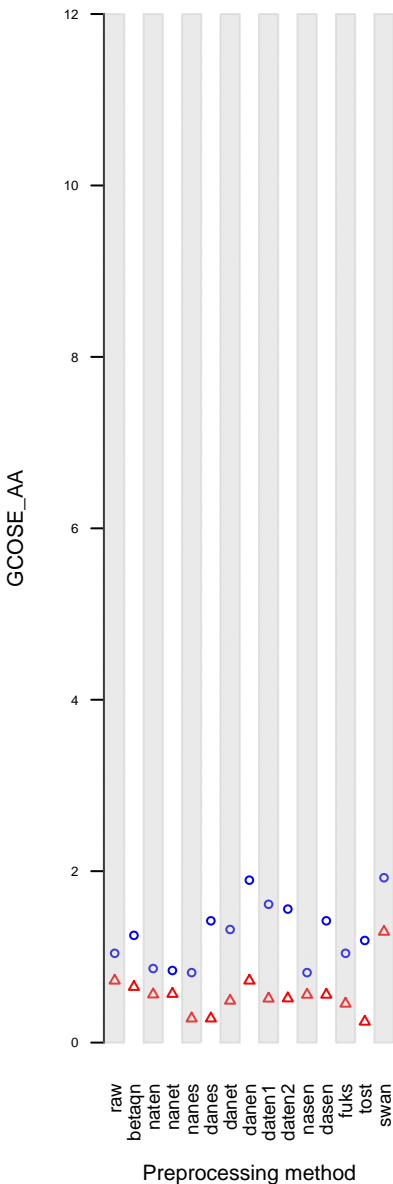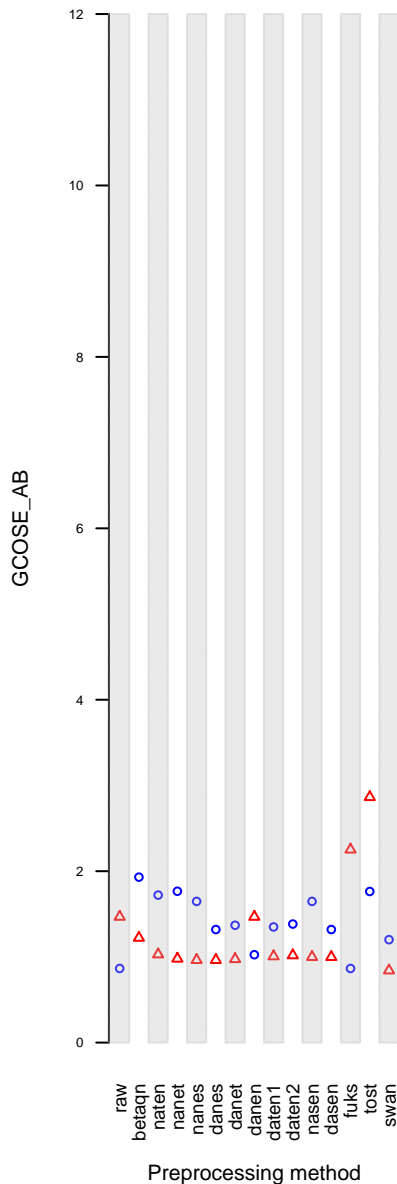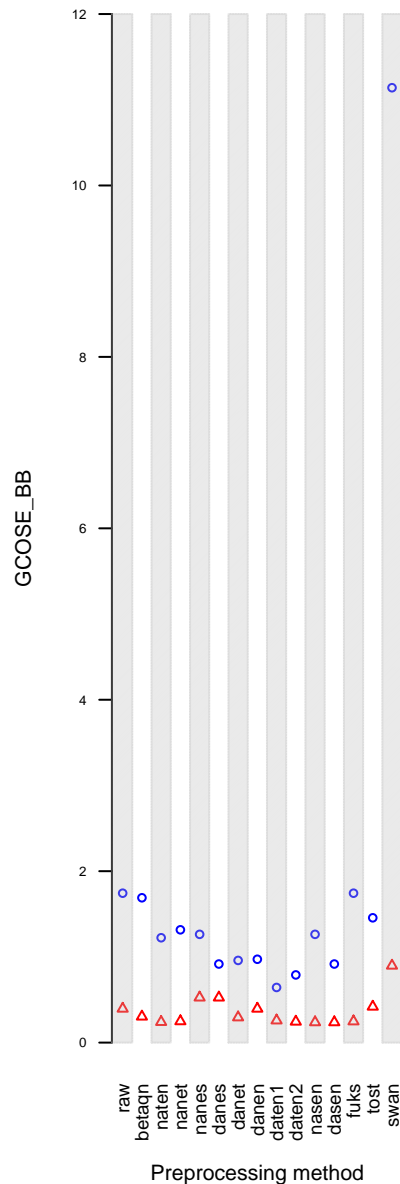

# Cohort\_1BC

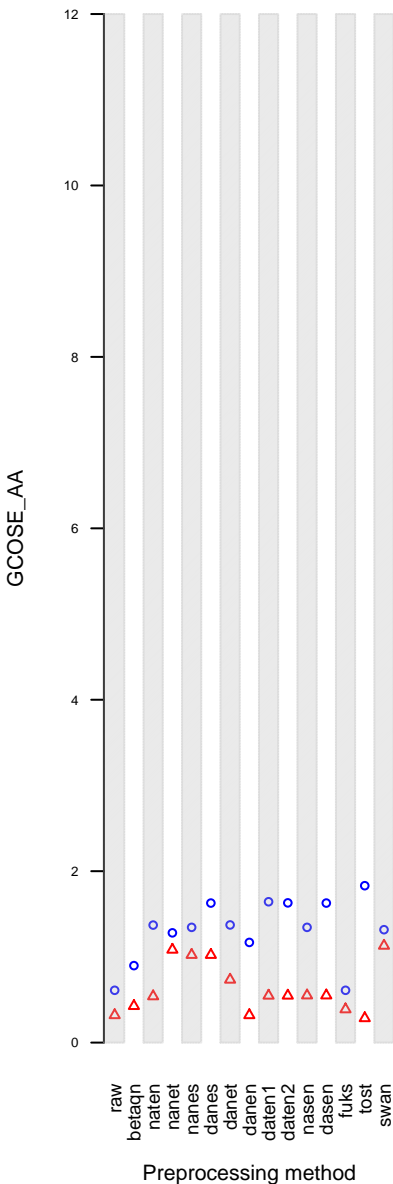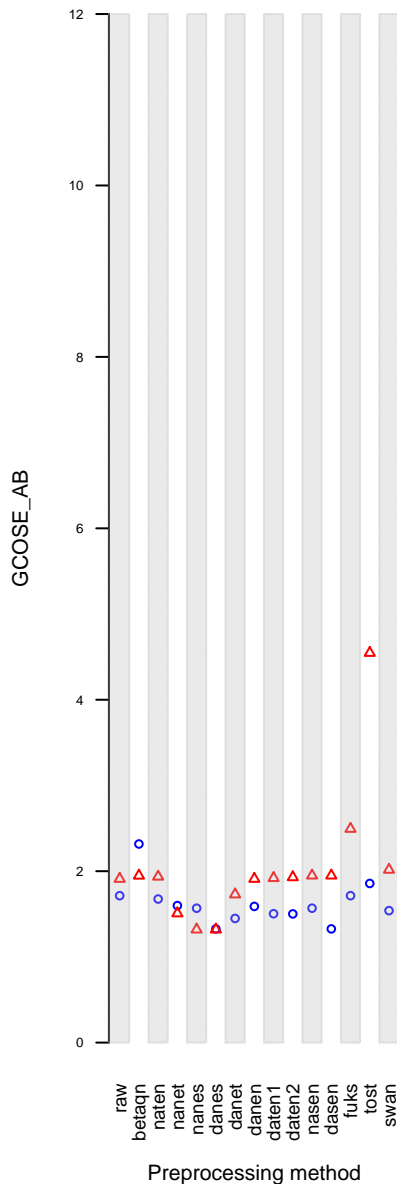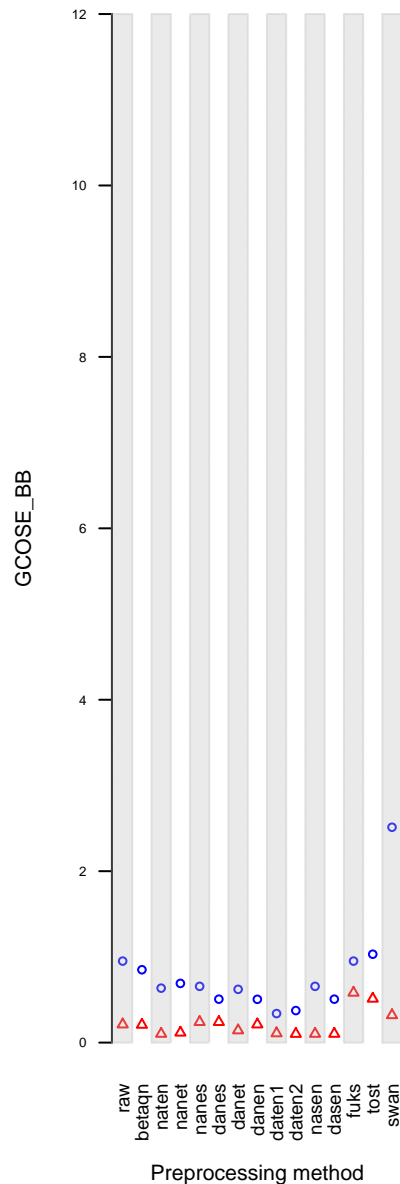

# Cohort\_1AD

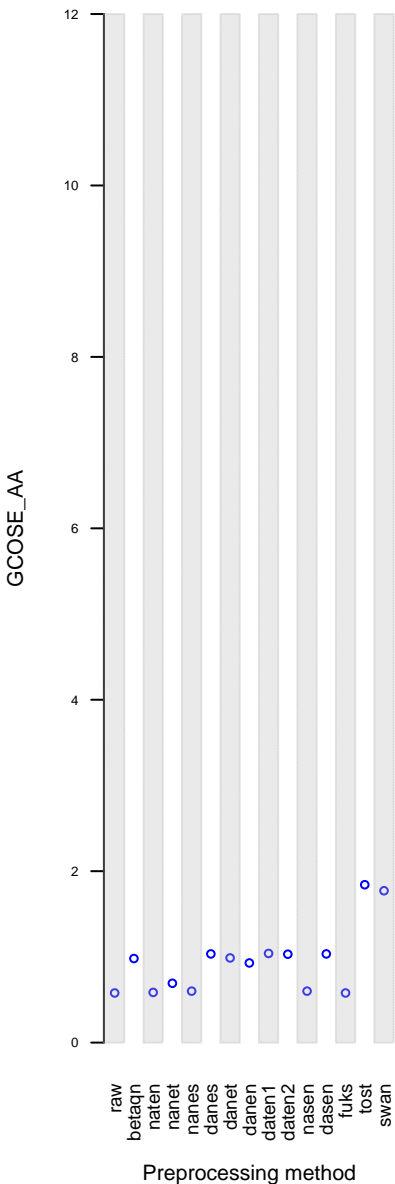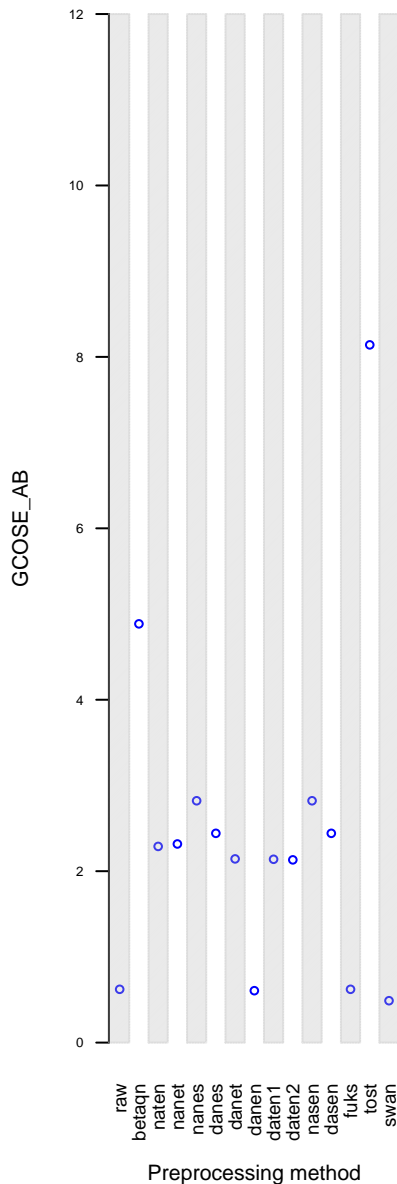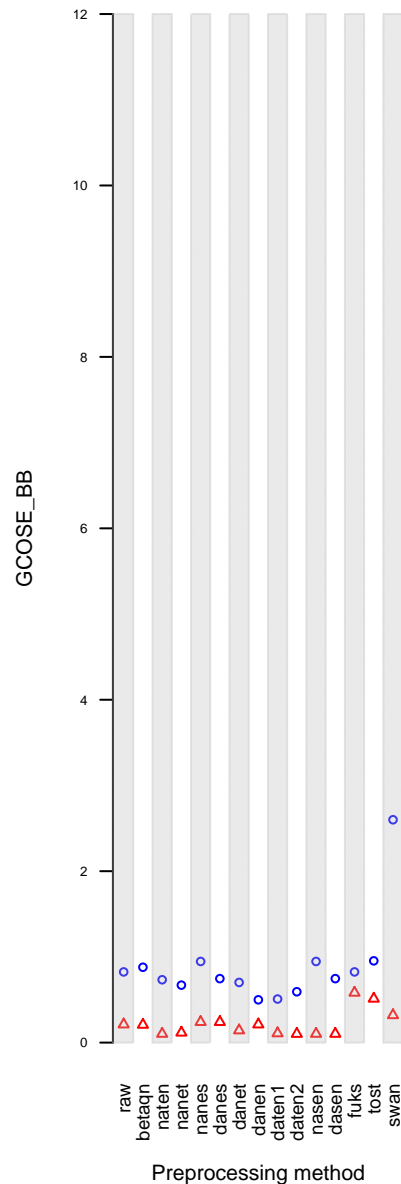

# Cohort\_1Aii

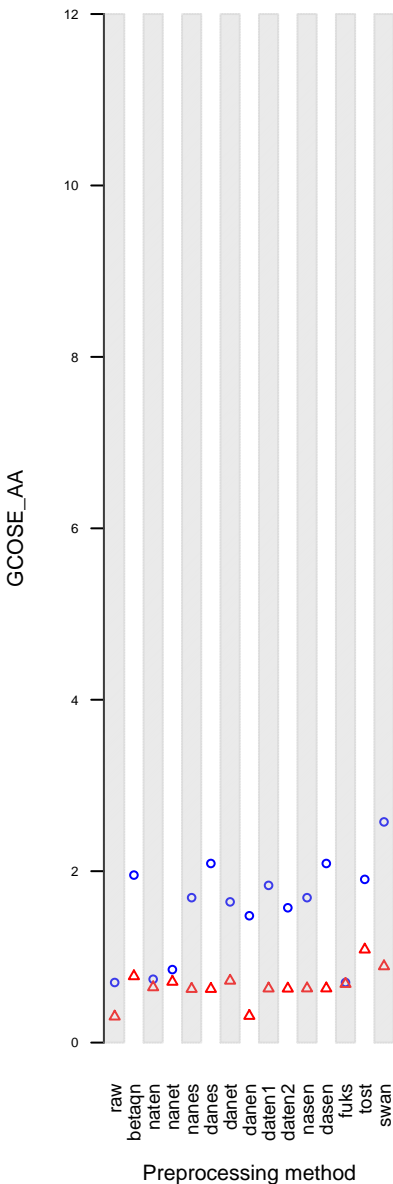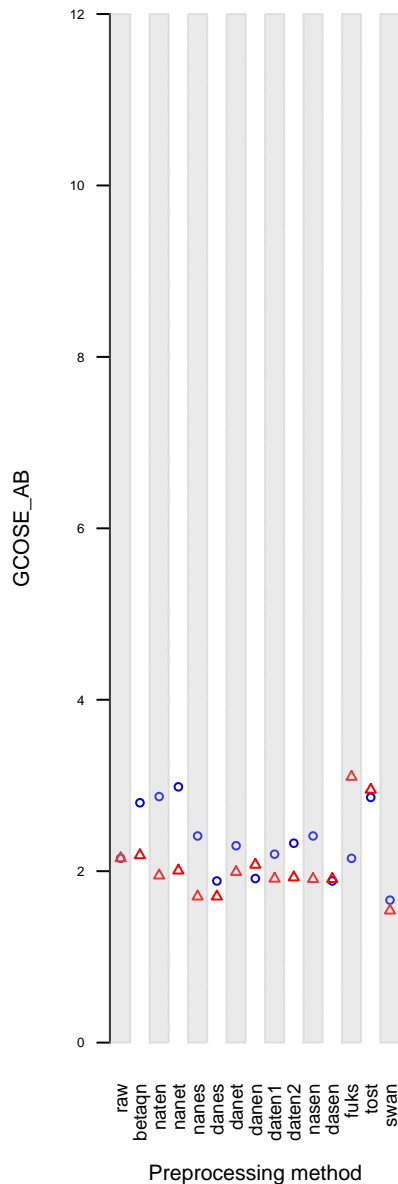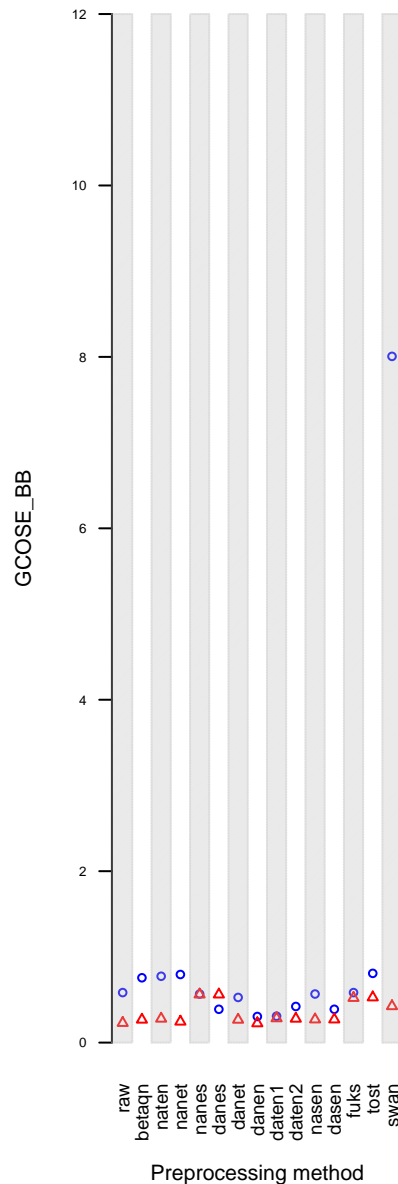

# Cohort\_1F

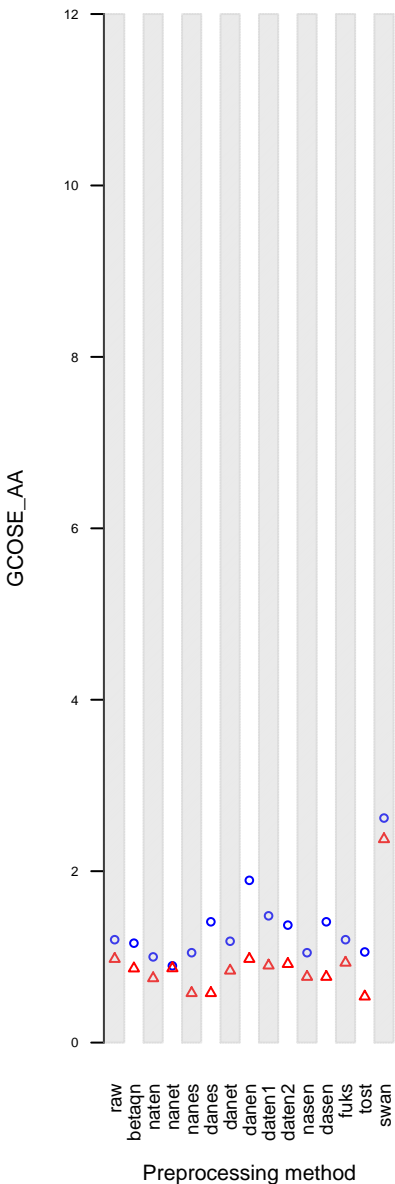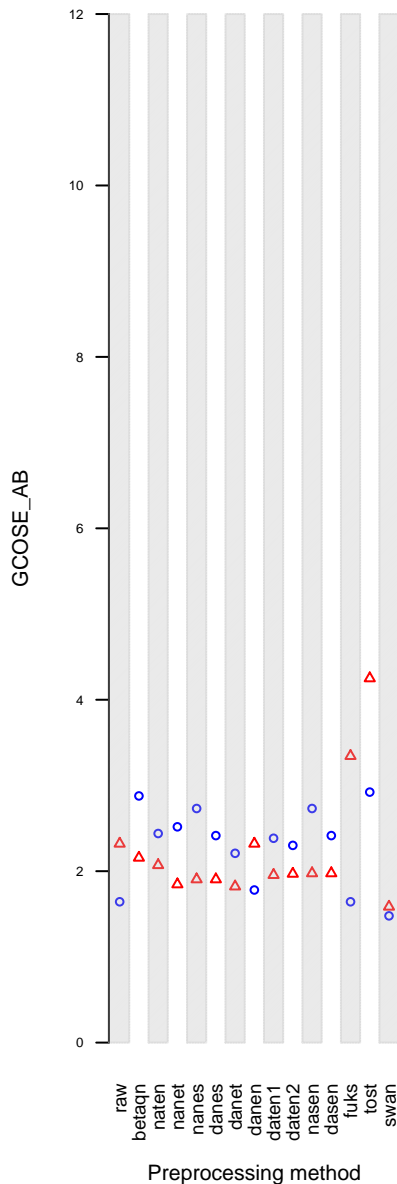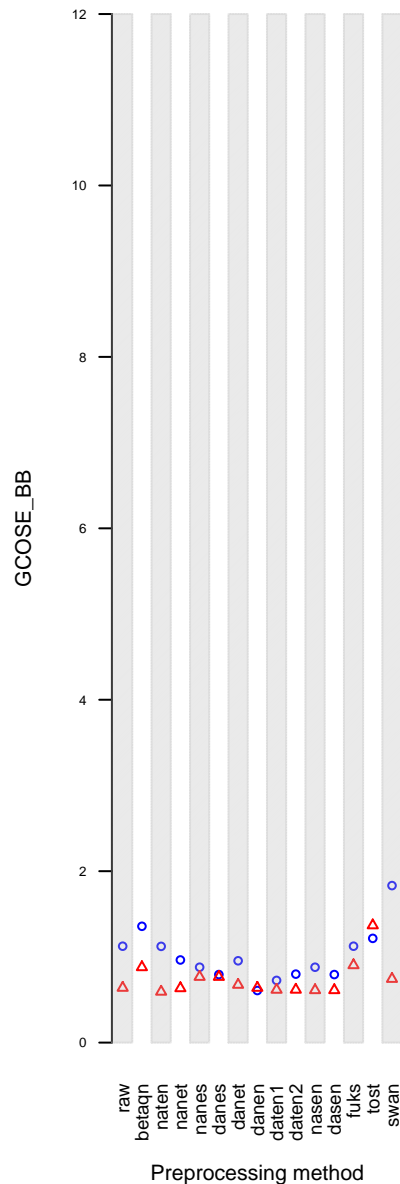

## Cohort\_2A

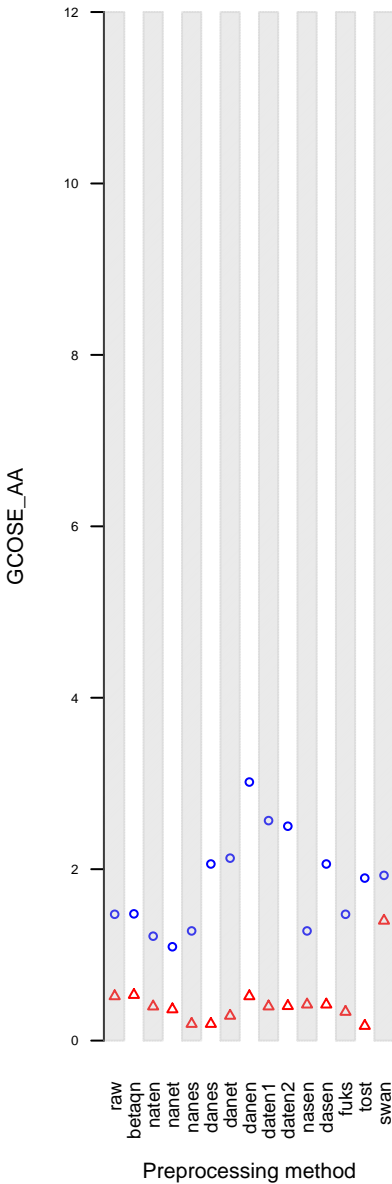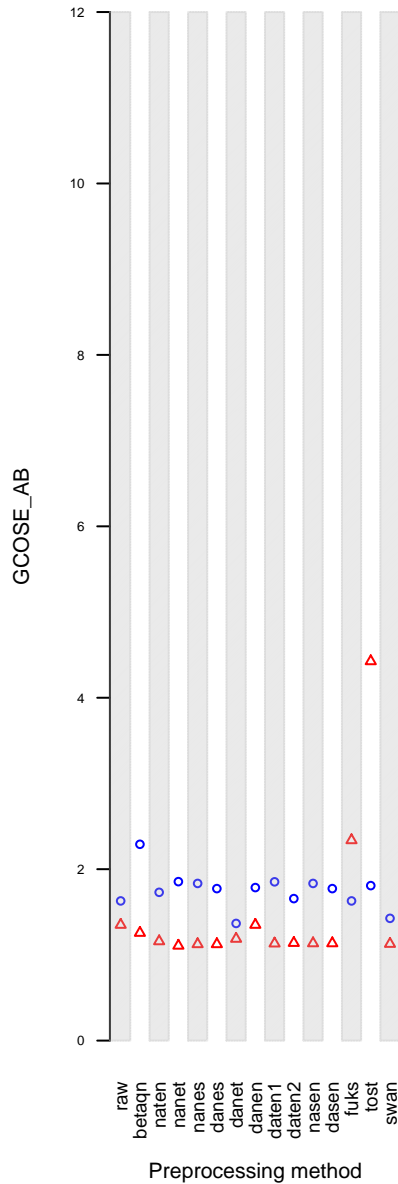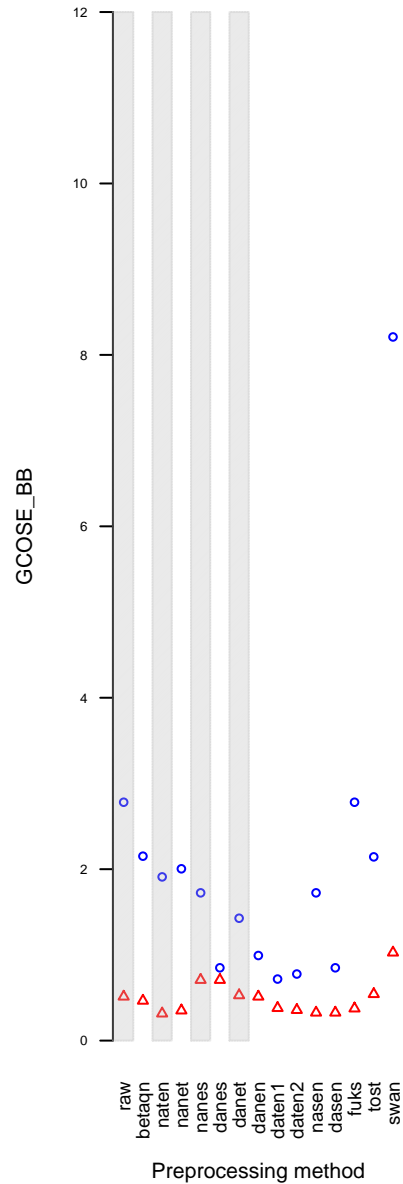

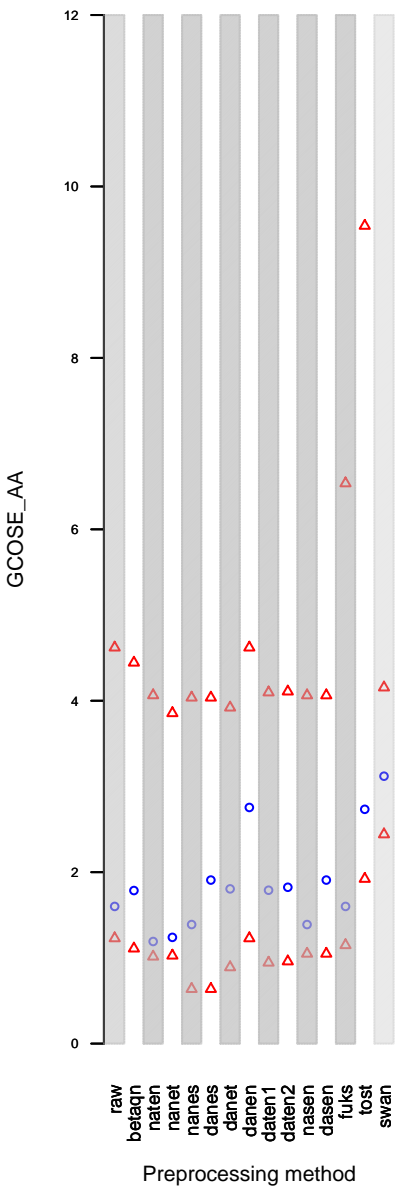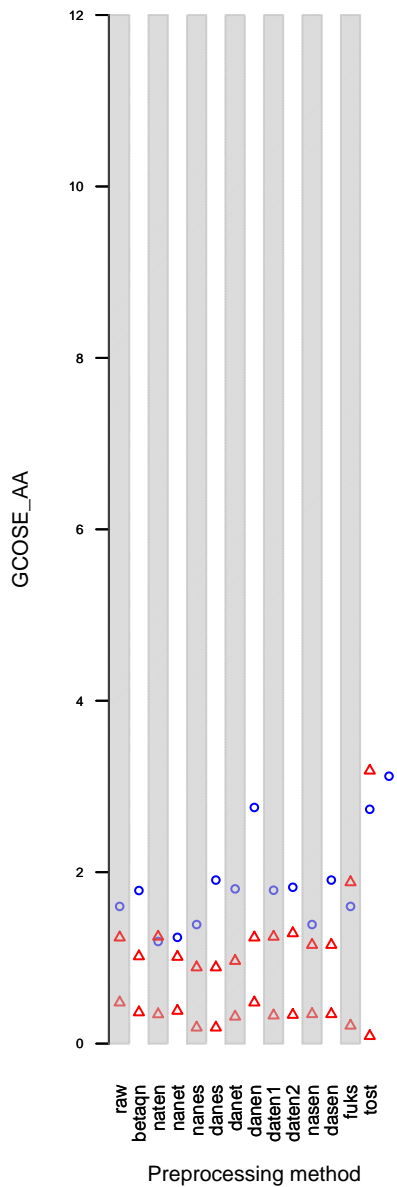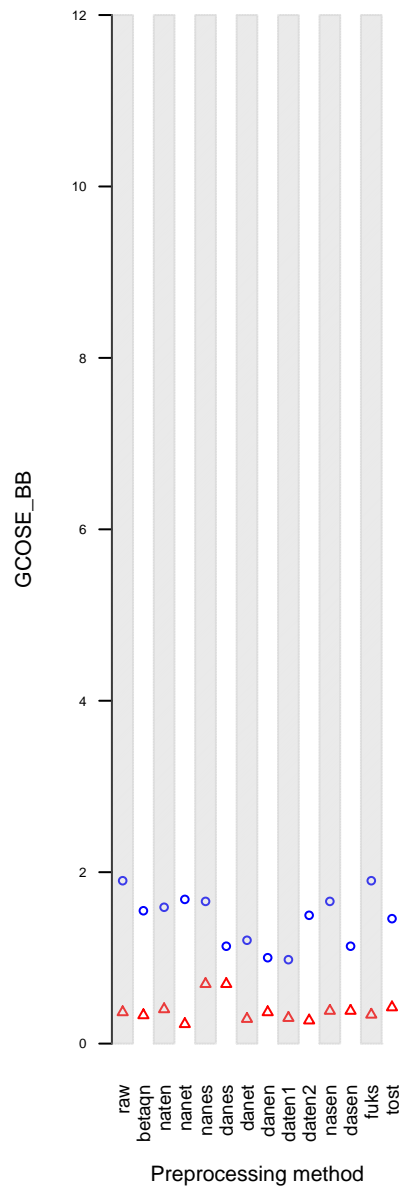

Supplement: Additional file 6 — Results of the GCOSE performance tests split by genotype group for all datasets (x10-4 scale is used on the y-axis). Lower values are indicative of a more sensitive preprocessing method. Type I probes are denoted by circles and Type II probes by triangles. This shows that the relative performance of our custom methods perform consistently across the range of betas, and that the tost method performs worst in the mid-range while swan does worst at the extremes. [file 1471-2164-14-293-S6.pdf]
