# Supplementary material for: A data-driven approach to preprocessing Illumina 450K methylation array data
Source: BMC Genomics. 2013 May 1;14:293. doi: 10.1186/1471-2164-14-293 (PMC3769145; doi:10.1186/1471-2164-14-293)
Supplement: Additional file 7 — Illustration of method ranking procedure. [file 1471-2164-14-293-S7.pdf]

Example of ranking method: Cohort_1BC, Type I probes

A. Calculate the metric scores (DMRSE, GCOSE_AA, GCOSE_AB, GCOSE_BB, Seabird) for each of the 15 preprocessing methods (raw…swan).

	raw	betaqn	naten	nanet	nanes	danes	danet	danen	daten1	daten2	nasen	dasen	fuks	tost	swan
DMRSE	2.22E-03	2.69E-03	1.98E-03	1.94E-03	1.45E-03	1.41E-03	1.87E-03	2.19E-03	1.91E-03	1.92E-03	1.45E-03	1.41E-03	2.22E-03	1.75E-03	2.15E-03
GCOSE_AA	6.10E-05	8.98E-05	1.37E-04	1.28E-04	1.34E-04	1.63E-04	1.37E-04	1.17E-04	1.64E-04	1.63E-04	1.34E-04	1.63E-04	6.10E-05	1.83E-04	1.32E-04
GCOSE_AB	1.71E-04	2.32E-04	1.68E-04	1.60E-04	1.57E-04	1.33E-04	1.45E-04	1.59E-04	1.50E-04	1.50E-04	1.57E-04	1.33E-04	1.71E-04	1.86E-04	1.54E-04
GCOSE_BB	9.51E-05	8.50E-05	6.35E-05	6.91E-05	6.56E-05	5.07E-05	6.22E-05	5.05E-05	3.39E-05	3.74E-05	6.56E-05	5.07E-05	9.51E-05	1.03E-04	2.51E-04
Seabird	4.68E-02	3.96E-02	3.82E-02	3.71E-02	4.72E-02	4.67E-02	3.75E-02	4.28E-02	3.80E-02	3.91E-02	4.72E-02	4.67E-02	4.68E-02	5.23E-02	5.00E-02

B. For each preprocessing method take mean of 3 SNP scores [A] to generate just 1 SNP score

	raw	betaqn	naten	nanet	nanes	danes	danet	danen	daten1	daten2	nasen	dasen	fuks	tost	swan
DMRSE	2.22E-03	2.69E-03	1.98E-03	1.94E-03	1.45E-03	1.41E-03	1.87E-03	2.19E-03	1.91E-03	1.92E-03	1.45E-03	1.41E-03	2.22E-03	1.75E-03	2.15E-03
GCOSE	1.09E-04	1.36E-04	1.23E-04	1.19E-04	1.19E-04	1.15E-04	1.15E-04	1.09E-04	1.16E-04	1.17E-04	1.19E-04	1.15E-04	1.09E-04	1.57E-04	1.79E-04
Seabird	4.68E-02	3.96E-02	3.82E-02	3.71E-02	4.72E-02	4.67E-02	3.75E-02	4.28E-02	3.80E-02	3.91E-02	4.72E-02	4.67E-02	4.68E-02	5.23E-02	5.00E-02

C. For each of the three metrics [B] rank the preprocessing methods

	raw	betaqn	naten	nanet	nanes	danes	danet	danen	daten1	daten2	nasen	dasen	fuks	tost	swan
DMRSE	13.5	15	10	9	3.5	1	6	12	7	8	3.5	2	13.5	5	11
GCOSE	2.5	13	12	11	9.5	5.5	4	1	7	8	9.5	5.5	2.5	14	15
Seabird	10.5	6	4	1	12.5	8.5	2	7	3	5	12.5	8.5	10.5	15	14

D. For each of the preprocessing methods calculate the mean of the 3 ranked metrics [C]

	raw	betaqn	naten	nanet	nanes	danes	danet	danen	daten1	daten2	nasen	dasen	fuks	tost	swan
Mean rank	8.8	11.3	8.7	7.0	8.5	5.0	4.0	6.7	5.7	7.0	8.5	5.3	8.8	11.3	13.3

E. For each preprocessing method calculate the mean of mean ranks [D] across the datasets, for Type I and Type II probes separately.

Type I

MEAN RANK	raw	betaqn	naten	nanet	nanes	danes	danet	danen	daten1	daten2	nasen	dasen	fuks	tost	swan
Cohort_1C [D]	5.17	11.67	9.00	8.67	7.50	4.83	8.67	5.33	7.00	10.67	7.50	4.83	5.17	11.00	13.00
Cohort_1B [D]	6.50	10.33	7.67	6.33	7.83	9.17	5.33	10.00	6.33	7.00	7.83	9.17	6.50	9.00	11.00
Cohort_1D [D]	4.17	11.33	9.33	7.67	6.50	6.00	8.67	7.00	7.67	10.00	6.50	5.67	4.17	10.67	14.67
Cohort_1Ai [D]	7.83	10.67	8.33	7.67	8.50	5.83	4.67	8.33	4.33	7.33	8.50	5.83	7.83	10.67	13.67
Cohort_1BC [D]	8.83	11.33	8.67	7.00	8.50	5.00	4.00	6.67	5.67	7.00	8.50	5.33	8.83	11.33	13.33
Cohort_1AD [D]	7.17	11.67	7.00	5.33	9.50	7.50	5.00	8.00	5.33	6.33	9.50	7.50	7.17	11.67	11.33
Cohort_1Aii [D]	5.83	14.33	9.67	9.00	9.50	4.50	8.33	5.33	6.33	6.00	9.50	4.50	5.83	11.33	10.00
Cohort_1E [D]	7.17	11.67	7.67	5.33	9.83	7.83	3.67	7.00	5.00	5.67	9.83	7.83	7.17	11.33	13.00
Cohort_2A [D]	7.50	14.33	11.00	10.67	4.17	5.83	4.33	7.00	6.00	6.33	4.17	5.83	7.50	14.00	11.33
Cohort_2B [D]	7.17	12.00	7.33	7.67	6.17	5.50	5.33	6.00	9.33	8.67	6.17	5.50	7.17	13.00	13.00
Cohort_3A [D]	11.50	12.00	9.00	11.00	5.50	3.00	4.00	8.00	3.00	5.00	5.50	3.00	11.50	13.00	NA
MEAN RANK OF RANK [E]	7.17	11.94	8.61	7.85	7.59	5.91	5.64	7.15	6.00	7.27	7.59	5.91	7.17	11.55	12.43

Type II

MEAN RANK	raw	betaqn	naten	nanet	nanes	danes	danet	danen	daten1	daten2	nasen	dasen	fuks	tost	swan
Cohort_1C [D]	9.67	11.67	9.67	7.67	7.50	7.50	6.00	10.00	5.00	5.67	1.50	1.50	11.33	13.33	12.00
Cohort_1B [D]	12.33	10.00	6.00	4.33	6.50	6.50	4.33	12.67	6.67	5.67	5.17	5.17	12.67	13.00	9.00
Cohort_1D [D]	12.33	11.00	7.67	5.67	7.83	7.83	7.00	12.67	6.00	5.67	3.50	3.50	10.33	11.00	8.00
Cohort_1Ai [D]	12.67	11.00	8.00	5.00	5.83	5.83	3.67	13.00	3.67	4.00	4.50	4.50	13.00	11.67	13.67
Cohort_1BC [D]	7.00	11.00	6.67	8.33	5.50	5.50	9.00	6.67	5.00	5.67	6.50	6.50	12.33	13.33	11.00
Cohort_1AD [D]	5.67	12.67	8.67	7.33	5.83	5.83	8.33	6.00	8.67	9.00	6.50	6.50	12.00	11.67	5.33
Cohort_1Aii [D]	10.00	12.33	7.00	4.67	9.83	9.83	8.00	9.67	5.00	4.67	5.17	5.17	10.00	9.33	9.33
Cohort_1E [D]	8.67	10.67	6.67	7.33	6.17	6.17	7.33	9.00	8.00	8.00	3.50	3.50	14.33	10.67	10.00
Cohort_2A [D]	13.67	11.00	4.67	3.67	8.50	8.50	3.67	12.67	3.67	4.00	4.50	4.50	14.00	12.00	11.00
Cohort_2B [D]	9.00	11.33	6.00	4.67	10.50	10.50	7.33	9.33	6.00	6.33	3.17	3.17	9.67	10.67	12.33
Cohort_3A [D]	12.00	7.00	9.50	4.00	4.00	4.00	3.00	12.00	6.50	8.50	4.50	4.50	13.50	12.00	NA
MEAN RANK OF RANK [E]	10.27	10.88	7.32	5.70	7.09	7.09	6.15	10.33	5.83	6.11	4.41	4.41	12.11	11.70	10.17

F. Rank the mean of metric values across all datasets [E] to generate a final score representing the performance of each of the 15 preprocessing methods (see Table 2).

[E]	Type I	Type II
raw	7.17	10.27
betaqn	11.94	10.88
naten	8.61	7.32
nanet	7.85	5.70
nanes	7.59	7.09
danes	5.91	7.09
danet	5.64	6.15
danen	7.15	10.33
daten1	6.00	5.83
daten2	7.27	6.11
nasen	7.59	4.41
dasen	5.91	4.41
fuks	7.17	12.11
tost	11.55	11.70
swan	12.43	10.17

Ranked scores

[F]	Type I	Type II	Average
raw	6.5	11.0	8.8
betaqn	14.0	13.0	13.5
naten	12.0	9.0	10.5
nanet	11.0	3.0	7.0
nanes	9.5	7.5	8.5
danes	2.5	7.5	5.0
danet	1.0	6.0	3.5
danen	5.0	12.0	8.5
daten1	4.0	4.0	4.0
daten2	8.0	5.0	6.5
nasen	9.5	1.5	5.5
dasen	2.5	1.5	2.0
fuks	6.5	15.0	10.8
tost	13.0	14.0	13.5
swan	15.0	10.0	12.5
